# Biological Effects of Korean Red Ginseng Polysaccharides in Aged Rat Using Global Proteomic Approach

**DOI:** 10.3390/molecules25133019

**Published:** 2020-07-01

**Authors:** Yong Yook Lee, Sung-Won Kim, Soo-Hyun Youn, Sun Hee Hyun, Jong-Su Kyung, Gyo In, Chae-Kyu Park, Hye Ryeon Jung, Seung Ju Moon, Min Jeung Kang, Eugene C. Yi, Chang-Kyun Han

**Affiliations:** 1 Laboratory of Fundamental Research, Korea Ginseng Corporation, 30, Gajeong-ro, Shinseong-dong, Yuseong-gu, Daejeon 34128, Korea; ace28@kgc.co.kr (Y.Y.L.); loveginseng@kgc.co.kr (S.-W.K.); soo@kgc.co.kr (S.-H.Y.); shhyun@kgc.co.kr (S.H.H.); jskyung@kgc.co.kr (J.-S.K.); 20109042@kgc.co.kr (G.I.); ckpark@kgc.co.kr (C.-K.P.); 2 Department of Molecular Medicine and Biopharmaceutical Sciences, School of Convergence Science and Technology and College of Medicine or College of Pharmacy, Seoul National University, Seoul 03080, Korea; hrj0523@snu.ac.kr (H.R.J.); msj0506@snu.ac.kr (S.J.M.); mjkang1104@gmail.com (M.J.K.); euyi@snu.ac.kr (E.C.Y.)

**Keywords:** Korean Red Ginseng, polysaccharide, hepatoprotective, immunity, anti-metastasis, proteomics, LC-MS/MS

## Abstract

Much has been written on the physiological benefits of Korean Red Ginseng (KRG). Among its various components, ginsenosides have been widely investigated for their various pharmacological effects. However, polysaccharides are a major KRG component that has not received scrutiny similar to that of ginsenosides. The present study aims to fill that gap in the existing literature and to investigate the possible functions of polysaccharide in KRG. The researchers evaluated proteomic changes in non-saponin fractions with rich polysaccharides (NFP) in KRG. Based on the serum analysis, proteomics analysis of the liver and the spleen was additionally conducted to identify related functions. We validated the suggested functions of NFP with the galactosamine-induced liver injury model and the cyclophosphamide-induced immunosuppression model. Then, we evaluated the antimetastatic potential of NFP in the lungs. Further proteomics analysis of the spleen and liver after ingestion confirmed functions related to immunity, cancer, hepatoprotection, and others. Then, we validated the suggested corresponding functions of the NFP in vivo model. NFP showed immune-enhancing effects, inhibited melanoma cell metastasis in the lung, and decreased liver damage. The results show that using the proteomic approach uncovers the potential effects of polysaccharides in KRG, which include enhancing the immune system and protecting the liver.

## 1. Introduction

Korean Red Ginseng (KRG) is processed Korean ginseng (*Panax ginseng* Meyer), a representative indigenous plant. KRG has been safely used as herbal medicine to normalize and strengthen body functions for a long time [1], making it a functional food. The representative activities of KRG are the boosting of immune function, helping in fatigue recovery, supporting blood circulation, acting as an antioxidant, improving memory function, and alleviating menopause symptoms [2]. The diverse pharmacological effects of KRG have been reported in both clinical and experimental research. These effects were the result of a combination of diverse components in KRG, namely, ginsenoside, non-saponin components such as polysaccharides, polyphenols, flavonoids, and others [3]. Among these components, ginsenosides have been well researched for various pharmacological activities. However, the activities of polysaccharides as major components of KRG have not been as deeply investigated as ginsenoside activities [4], because ginsenosides seemed easier to isolate and characterize in terms of chemical structure. For this reason, polysaccharides have been less explored, in terms of evaluating all their possible functions individually.

A proteomic research strategy presents comprehensive biological data from changes in related proteins, suggesting their interrelationships [5]. In a previous study, we demonstrated a proteomics research tool to study the bioactivity and biomarkers of KRG [6]. Currently, multiomics strategy systems are applied in the field of natural product studies [7,8,9,10].

The present study aims to investigate and identify the effects of polysaccharides in KRG. We evaluated proteomic changes in non-saponin fractions with rich polysaccharide (NFP) in KRG between a blank group (control group) and an NFP-treated group, in order to investigate how polysaccharides work in KRG. A label-free global proteomic analysis was employed to compare proteins between the groups in serum, in order to identify NFP’s effects on human physiology. Based on these results, the possible effects of NFP were identified and selected for testing in relation to organ efficiency. Then, we investigated several effects of the NFP in order to validate the suggested functions of the NFP in vivo model.

## 2. Results

### 2.1. Protein Identification for Serum Proteins

**Figure 1:**
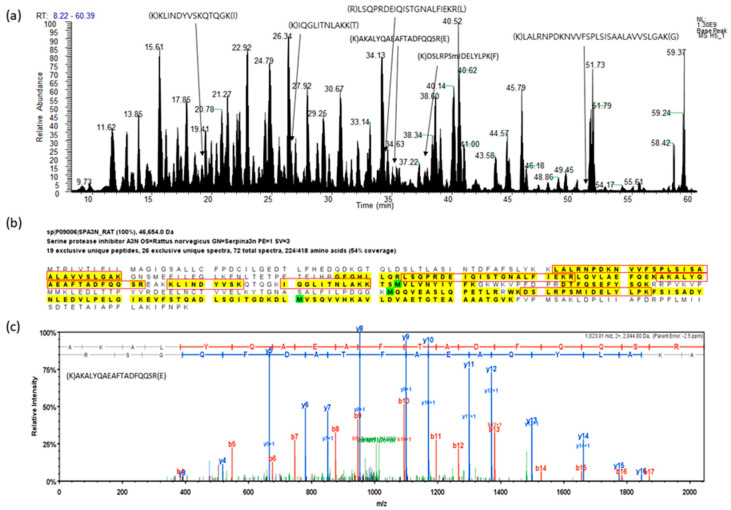
A representative example of the analysis for fractionated serum protein digest by liquid chromatography with tandem mass spectrometry (LC-MS/MS). (**a**) Base peak chromatogram based on total ion chromatography, the peptides indicate identified peptides of SPA3N. (**b**) The sequence of SPA3N, the peptides in the red boxes indicate peptides found by MS/MS. (**c**) The experimental MS/MS spectrum for the SPA3N doubly charged tryptic peptide sequence AKALYQAEAFTADFQQSR among the identified peptides by matching a peptide fragmentation pattern.

The researchers performed a proteomic analysis of rat sera to identify differentially expressed proteins (DEPs) in the first step. The representative protein identification process and spectra are provided in Figure 1. We compared the total identified protein list at 1% false discovery rate (FDR) level (Supplemental Table S1), and selected proteins with the following criteria to identify the proteins under a stringent level of evaluation: *p* < 0.05 (T-test), and *p* < 0.025 (PLGEM *p*-value), as shown in Table 1. PLGEM here is the power law global error model. 

Then, we performed enrichment analysis using the Database for Annotation, Visualization, and Integrated Discovery (DAVID) for functional annotation, to estimate the relevant biological function. For example, Gstm1, Gstm2, and Gsta3 as upregulated proteins showed a function associated with glutathione and redoxin metabolism. Meanwhile, proteins related to hepatitis B, viral carcinogenesis, and platelet aggregation were all shown to have decreased. In the next step, we enlarged the total pool of proteins (protein: probability > 99.9%; peptide: probability > 95%) (Supplemental Table S3) for bioinformatics evaluation, using an Ingenuity Pathway Analysis (IPA) tool in three different conditions: (1) all organs and cell lines for the overall changes; (2) focusing on cancer-related functions and cell lines, because cancer-related bio functions were mainly identified via existing criteria; and (3) other organs and cell lines except cancer-related functions. Our particular interest was to identify all the possible effects of NFP, including new functions. As a result, the overall upregulated functions we identified were related to the activation of cells (phagocytes and macrophages), and the number of cells (Table 2). Meanwhile, various cancers, brain diseases, viral infections, and the synthesis of reactive oxygen species were downregulated. In the cancer-specific evaluation (criteria 2), the IPA analysis showed that the proteins were related to cancer types and anticancer functions, like metastasis, cell spreading, and angiogenesis. Based on the results from the IPA analysis, we further evaluated organs, like the spleen, for immunity and cancer, as well as the liver for antioxidants and liver protection. 

### 2.2. Protein Identification: Spleen and Liver as Select Target Organs

Based on the analysis of serum proteins, we selected the liver and the spleen as primary candidate organs. In the next step, we further compared the proteomic profiles between control and NFP. A total of 4656 unique proteins were identified by the MS analysis of the spleen, and 3583 for the liver (protein: probability > 99%; peptide: probability > 95%) (Supplemental Table S3).

Among the DEP pool (*p* < 0.001), between the normal and the NFP, we selected the DEPs whose changes showed top upregulated/downregulated proteins in Table 3. 

The DEPs in the spleen showed a function associated with innate immune response, biosynthesis of antibiotics, mitochondria, and amoebiasis derived using DAVID. At the same time, the liver DEPs were related to the glutathione metabolic process and wound healing. Then, we integrated the DEPs into the context of biological processes, using the same approach as that used for the IPA in the serum analysis. Our analysis of the results from the IPA tool revealed that the movement of immune cells was upregulated in the analysis of the spleen. In contrast, the cell death of hepatocytes, liver necrosis, and inflammation were downregulated in the liver (Table 4).

### 2.3. A More Detailed Function Analysis of the Proteins

**Figure 2:**
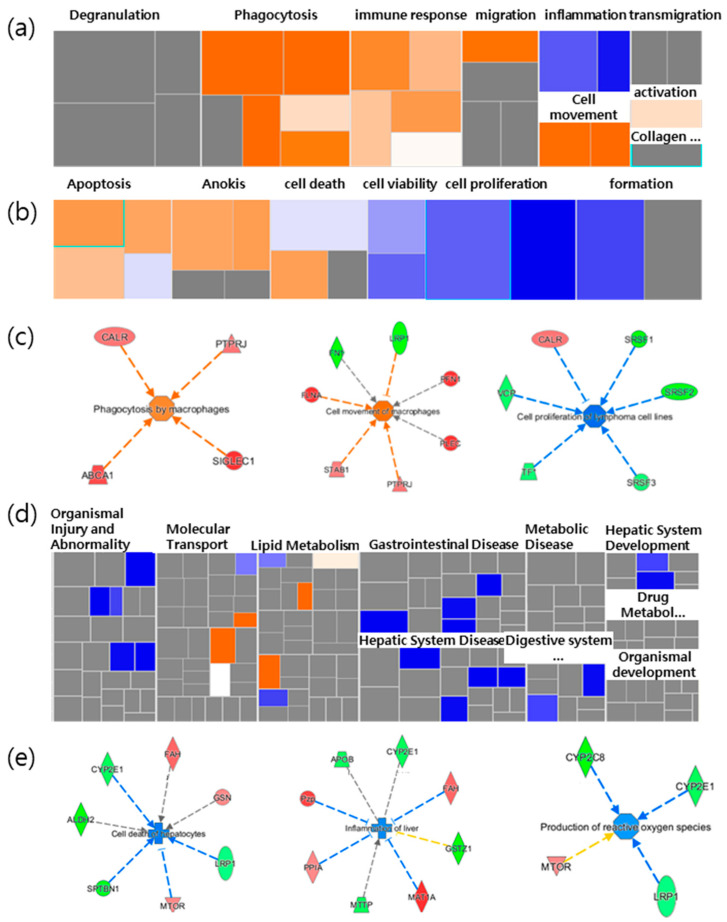
Heatmap analysis of selected functions related to spleen and liver. The heatmap showed (**a**) inflammatory response related to immunological functions and (**b**) cell death and survival related to cancer in the spleen. (**c**) Selected increased immunological functions, and the cell death and survival function of cancer in the spleen. (**d**) The heatmap panel shows hepatic functions in the liver. (**e**) Selected decreased typical function in the liver led to cell death of hepatocytes, inflammation of the liver, and the production of reactive oxygen species (ROS) in the liver. Each box represents a biological process or disease. The color of the box (**a**,**b**,**d**) and the proteins (**c**,**e**) indicates the predicted increase or decrease. Orange boxes represent biological processes or diseases that are trending toward an increase. Blue boxes represent biological processes or diseases that are trending toward a decrease. Grey boxes represent biological processes or diseases that are not predictable (currently ineligible for a prediction). NFP is a non-saponin fraction with rich polysaccharide.

Based on the results from the IPA, we focused on the main bio functions related to both the spleen immune function and the liver-protective function. The DEPs of the spleen were mainly related to cancer, and inflammatory responses related to immune functions. A more detailed analysis of the inflammatory response-related immune functions revealed that movement, migration, and phagocytosis increased with immune cells, as shown in Figure 1a. For the functions associated with cell death and the survival of cancer, the apoptosis of tumor cells and carcinomas, anoikis, and cell death were all shown to increase. In contrast, cancer cell proliferation decreased (Figure 2b). Thus, the results showed that NFP was involved in cancer cell proliferation while increasing the immune response (Figure 2c). 

Concerning hepatic functions, it was mostly gastrointestinal disease that was downregulated (Figure 1d). The cell death of hepatocytes and inflammation were reduced, while reactive oxygen species (ROS) and lipid synthesis were also slightly decreased. For example, the fumarylacetoacetate hydrolase (Fah) gene increased, and Cype2e1 decreased, in relation to both the cell death and inflammation functions. In addition, there was a decrease in Stopn1 and low-density lipoprotein receptor-related protein 1 (Lrp1), and increases in the pregnancy zone protein (Pzp), peptidylprolyl isomerase A (Ppia) and methionine adenosyltransferase 1A (Mat1a), which were associated with decreases in inflammation (Figure 1e). These results implied that the NFP has a protective effect on liver tissue and cells. Based on the results, we evaluated the effects of the enhanced immune function of immunoglobulin M (IgM) antibody-producing cells, the antimetastatic potential in the lungs, as well as liver protection and antioxidants, with the in vivo model as the validation target. 

### 2.4. IgM Antibody-Producing Cells in the Spleen

**Figure 3:**
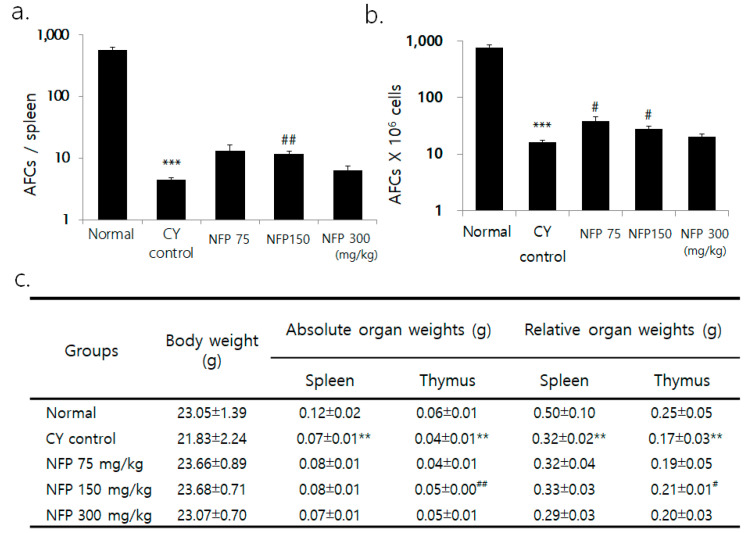
Effects of NFP on antibody-forming cells (AFCs) and splenic subpopulation in Bagg Albino (BALB/c) mice treated with cyclophosphamide (CY). (**a**) Decrease in AFCs/spleen from the normal group at *** *p* < 0.001, increase in AFCs/spleen in the NFP 150 mg/kg groups compared with the CY control at ^##^ *p* < 0.01. (**b**) Decrease in AFCs (× 10^6^) cells from the normal group at *** *p* < 0.001, increase in AFCs (× 10^6^)/spleen in the NFP 75, 150 mg/kg-treated groups compared with the CY control at ^#^*p* < 0.05. (**c**) Comparing the normal group and the CY control group shows a decrease of **p < 0.01. However, in the NFP group administered with 150 mg/kg, the absolute organ weight (^##^ *p* < 0.01) and relative organ weight (^#^ *p* < 0.05) of the thymus increased. The data represent means ± standard error of the mean (SEM) (*n* = 8).

Although the researchers observed no significant difference in body weight between the groups, the weight of the spleen and thymus decreased in the cyclophosphamide (CY) group for immunosuppression, compared to the normal group (Figure 3c). Compared to the CY control, the thymus weight was greater in the group administered with 150 mg/kg of NFP (*p* < 0.01). Across all splenic cells, the count of plasma cells significantly decreased in the CY control compared to the normal group, confirming CY-induced immunosuppression (*p* < 0.001). NFP administration resulted in a trend of increased plasma cell count, especially with the administration of 150 mg/kg, with a statistical significance (*p* < 0.01), as in Figure 3a. The plasma cell count per 1 × 10^6^ splenic cells significantly decreased in the CY control, compared to the normal group. Meanwhile, 75-mg/kg and 150-mg/kg of NFP resulted in an increased plasma cell count, with statistical significance (*p* < 0.05), as in Figure 3b. 

### 2.5. Evaluation for the Antimetastatic Potential of NFP in the Lungs

**Figure 4:**
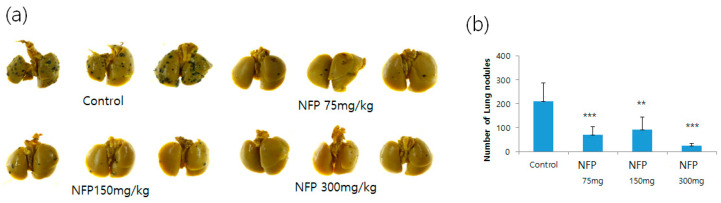
Effects of NFP on the lung metastasis of B16F10 melanoma cells in vivo. (**a**) Photographs of lung colonization in C57BL/6 mice orally administered with NFP at doses of 75, 150 and 300 mg/kg/day, compared with the vehicle control group. (**b**) Quantitative analysis of lung nodules of B16F10 melanoma cells. Data represent the mean-standard deviation (SD) (*n* = 6). Statistical significance of differences between the vehicle control and NFP treatment groups was evaluated using Student’s t-test (* *p* < 0.05, ** *p* < 0.01, *** *p* < 0.001).

NFP was orally administered to mice intravenously injected with B16F10 cells, to confirm that NFP could inhibit the formation of distant metastasis in vivo. The NFP treatment for mice intravenously injected with B16F10 cells resulted in increased inhibition of lung metastasis, compared with the vehicle control (Figure 4a).

As shown in Figure 4b, the number of metastatic colonies was counted. In the vehicle control without the NFP treatment, 210 ± 77 metastatic colonies were generated. In contrast, the corresponding colony numbers in mice orally administered with 75, 150 and 300 mg/kg/day of NFP were 70 ± 33 (*p* < 0.001), 91 ± 52 (*p* < 0.01) and 23 ± 10 (*p* < 0.001), respectively.

### 2.6. Galactosamine (GalN)-Induced Rat for Liver Injury Model

**Figure 5:**
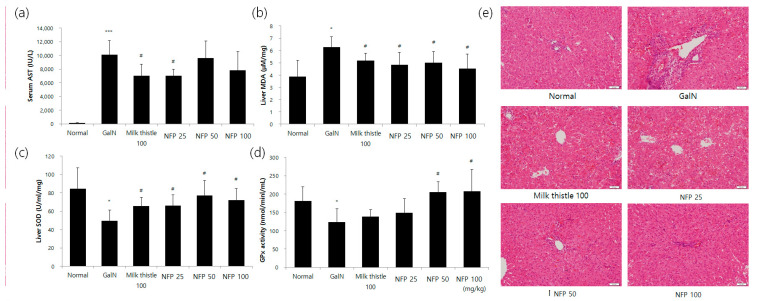
Effects of NFP on liver functions, oxidative stress markers, and histopathological changes (GalN, 400 mg/kg, single intraosseous (IP) dose). (**a**) Decreased aspartate transaminase (AST) activity in the NFP 25 mg/kg groups compared with the GalN control at ^#^ *p* < 0.05, *** *p*< 0.001. (**b**) Decrease in the lipid peroxidation product malondialdehyde (MDA) in the NFP 25, 50, and 100 mg/kg groups, compared with the GalN control at ^#^ *p* < 0.05. (**c, d**) Increase in the superoxide dismutase (SOD) (25, 50, and 100 mg/kg) and GPx (50 and 100 mg/kg) levels in the NFP groups compared with the GalN control at ^#^ *p* < 0.05. (**e**) NFP showed an absence of ballooning, inflammatory cells, regeneration of hepatocytes around the central vein, and also a slight congestion in the central vein. Data represent means ±SD (*n* = 8). Magnification: 20×, Scale bar: 50 μm.

The results regarding serum aspartate transaminase (AST) levels are shown in Figure 5. In the GalN-treated group, serum AST levels were significantly increased, compared with those in the normal group. The AST levels seemingly tended to decrease with the concentration of NFP treatment. The alanine transaminase (ALT) level was reduced via treatment using 25 mg/kg NFP, with significance (*p* < 0.05) similar to that of the milk thistle, which was used as a positive control (Figure 5a). Hepatic malondialdehyde (MDA), superoxide dismutase (SOD) and glutathione peroxidase (GPx) activities were measured to evaluate the antioxidant activity of the liver. Treatment with NFP of 25, 50 or 100 mg/kg, and milk thistle (100 mg/kg), significantly recovered MDA and SOD levels as the antioxidant parameters of liver damage (*p* < 0.05), as shown Figure 5b,c.

The NFP showed an increasing pattern of GPx activity (Figure 5d). The NFP treatments with 50 and 100 mg/kg were shown to increase the GPx activity with statistical significance (*p* < 0.05). For histopathological examination, we performed the hematoxylin and eosin (H&E) staining study in the different treatment groups. Compared with the control group, the GalN-challenged group showed an inflammatory response. The NSP showed the absence of ballooning and inflammatory cell infiltration (Figure 5e). 

## 3. Discussion

Many KRG-related articles look at its active components, like ginsenoside and polysaccharide, but polysaccharides have not been as deeply investigated when compared with ginsenosides [4]. With this in mind, this study aimed to fill this gap in the literature. In the present study, we prepared an NFP of KRG. It is widely accepted that older adults are more susceptible to various diseases compared to young adults [11] because biological aging comes with increased morbidity and multiple function decline [12]. Thus, aged animal models seemed suitable for assessing the diverse activity of NFP. 

The researchers performed a comprehensive serum proteomic analysis to compare proteins between the NFP-treated groups and the nontreated group in old rats, using bioinformatic interpretation to identify the potential activities of NFP. As a result, a variety of functions were identified that are related to the activation of immune cells, brain disease, viral infection, liver-related functions, synthesis of reactive oxygen species, and other effects (Table 2). In addition, the cancer-specific analysis showed that the proteins were related to types of anticancer, anticancer functions (including metastasis), cell spreading, and angiogenesis. Based on the results, we performed an additional analysis of the spleen to test the NFP’s effect in relation to immune function and cancer, and another analysis of the liver in relation to antioxidants and liver protection. 

Proteomic analysis of the spleen suggested that the NFP increases immune cell activity by increasing cell movement and migration. In addition, it is thought to increase innate immunity by increasing phagocytosis and internalization. In terms of anticancer activity, NFP is expected to induce apoptosis and carcinoma death, while downregulating the proliferation and survival rate of cancer cells. The top regulated DEPs in the spleen are listed in Table 3 and Table 4. Sialic acid-binding immunoglobulin-type lectins (Siglecs) are cell-surface proteins that bind sialic acid, which, in turn, binds the proteins of type A and B influenza viruses [13]. It has been reported that Siglecs are involved in the phagocytosis of bacteria, while some Siglecs are capable of phagocytosing pathogens that express cell-surface sialic acid moieties. Siglec1 is a member of the Siglec family, expressed on a number of macrophage populations. Siglec1 has been reported to mediate phagocytosis of the Gram-negative bacteria [13,14]. Fn1 (fibronectin) is a component of the extracellular matrix that binds to integrins, and plays important roles in cell adhesion, migration, growth and differentiation [15]. Fn1 was also reported as a major component during wound healing [16]. Plec is a protein that acts as a cytoskeletal linker protein, which plays an important role in maintaining tissues, cell integrity, coordinating dynamic alterations in cell shape, and cytoarchitecture [17,18]. Plec was also reported as essential to the integrity of muscle cells in epithelial cells [19], and vital to the crosslinking of actin filaments and microtubules in the nervous system [20]. The protein serine and arginine-rich splicing factor 1 (Srsf1) is a proto-oncogene [21] that can act as an oncoprotein, and is an important target for cancer therapy, as it is overexpressed in many tumors [22]. Extracellular matrix molecules, including fibronectin, have been identified as activators of the toll-like receptors that function as regulators of the innate immune system in response to pathogens and damaged tissue [23]. Filamin alpha (Flna) is a multifunctional protein that binds to integrins and couples them to the actin cytoskeleton [24]. Flna plays an important role in infection by viral pathogens [25,26]. However, little is known about its interactions with components of the host’s innate immune response [27].

Based on the results, we evaluated the NFP’s effect on immunity using IgM antibody-producing cells in the spleen. NFP significantly increased the number of antibody-forming cells in the spleen of the CY model. Therefore, we confirmed that NFP exhibited immunity-enhancing effects. For the immune functions of the polysaccharides, it was reported that polysaccharides from ginseng or red ginseng could activate macrophage functions through toll-like receptor 2 (Tlr2) and stimulate macrophages to produce Th1 and Th2 cytokines. Furthermore, polysaccharides stimulate the dendritic-cell-enhancing production of interferon-γ, thereby promoting the production of cytotoxic cells against tumors. The present study also reported that KRG polysaccharides enhance the phagocytic activity of macrophages, which was consistent with our analysis [28,29]. 

In addition, we evaluated the effects of NFP on the lung metastasis of B16F10 melanoma cells in vivo. The results showed that NFP significantly inhibited melanoma cell metastasis in the lungs. It was reported that lung metastasis in B16-F10 melanoma-bearing mice was reduced significantly by intraosseous (IP) [30] and per os (PO) [31] administration of red ginseng acidic polysaccharide. 

Liver disease is regarded as a global health problem [32]. One of the solutions that researchers have looked into is the possible beneficial effects of KRG on liver disease. Some studies have reported that KRG has hepatoprotective effects against hepatotoxins like hydrogen peroxide, alcohol, carbon tetrachloride, aflatoxin B1, diethylnitrosamine, viruses, and inflammation. Moreover, KRG has antioxidative effects in nonalcoholic fatty liver disease (NAFLD) [33]. Most studies have been conducted with ginsenosides, and only a few have studied the effects of polysaccharides on CCl_4_-induced hepatic injury [34] and hepatocellular carcinoma [35]. Some studies reported that there are correlations between NAFLD and obesity, dyslipidemia, and metabolic syndrome [36,37]. Thus, the present study looked into the hepatoprotective effects of KRG polysaccharides and their beneficial effects on various liver-related diseases. 

A detailed liver proteomic analysis of the IPA function showed the possibility of NFP reducing hepatocyte cell death and liver inflammation, and inhibiting ROS production and synthesis. The top regulated DEPs in the liver are listed in Table 3 and Table 4. Glutathione, as an endogenous antioxidant, plays a key role in the maintenance of the intracellular redox balance, and the detoxification of xenobiotics [38]. The DAVID analysis showed that glutathione peroxidase 1 (Gpx1), Glutathione S-transferase alpha 1 (Gsta1), Glutathione S-transferase Mu 2 (Gstm2) and hemoglobin subunit beta (Hbb) are glutathione metabolic process proteins. Gpx1, as an antioxidant enzyme counteracting oxidative stress, is ubiquitously expressed in many tissues, where it plays an important role in modulating intracellular ROS [39]. HBB is a globin protein, along with alpha globin (Hba). Its functions involve oxygen transport from the lungs to various peripheral tissues [40]. Meanwhile, Aldh2 is a mitochondrial enzyme that is highly expressed in the liver, and plays a crucial role in the detoxification of reactive acetaldehydes [41]. Methionine adenosyltransferase (Mat) is the enzyme responsible for the synthesis of S-adenosyl-L-methionine, a biological methyl donor required for methylation [42]. There are three types of genes that encode protein products: Mat1a, Mat2a and Mat2b [43]. Mat1a is expressed only in adult hepatocytes, whereas Mat2a shows a wider distribution [44]. There are also reports linking Mat and liver dysfunction, which detailed that patients with advanced NAFLD exhibit Mat1a hypermethylation and lower Mat1a mRNA levels, compared to patients with mild NAFLD and normal subjects [45]. In addition, the livers of Mat1a-deficient mice exhibit increased oxidative stress caused by low glutathione levels [46]. Hepatoprotective and antioxidant effects were confirmed in the GalN-induced rat for the liver injury model.

We plan to analyze brain-related functions, one area of the suggested functions in serum proteomics. In this research, we suggested the overall potential activities of polysaccharides in KRG. Among these activities, we confirmed how polysaccharides, or NFP, have immunity-enhancing and liver-protective effects. 

## 4. Materials and Methods

### 4.1. Preparation of the KRG Water Extract and General Chemicals

The KRG concentrates (15 brix) from six-year-old *P. ginseng* (Meyer) root were obtained from the Korea Ginseng Corporation (Buyeo, Republic of Korea). Carbohydrate-digesting enzymes, such as α-amylase from *Aspergillus oryzae*, amyloglucosidase from *Aspergillus niger*, and pectinesterase from orange peels, were all obtained from Sigma (St. Louis, MO, USA). Meanwhile, polygalacturonase from *Aspergillus aculeatus* was purchased from Megazyme (Bray, Ireland). On the other hand, ammonium bicarbonate (NH_4_HCO_3_), dithiothreitol (DTT), formic acid (FA), iodoacetamide (IAA) trifluoroacetic acid, ammonium formate, and urea were purchased from Sigma-Aldrich (St Louis, MO, USA). The HPLC-grade acetonitrile (ACN) and water were purchased from JT Baker (JT Baker, Phillipsburg, NJ, USA). Lyophilized trypsin was obtained from Promega (Madison, WI, USA). 

### 4.2. Extraction of the NFP Fraction

The extraction procedure for the NFP from the KRG concentrate was carried out via ethanol precipitation, enzyme hydrolysis, and size exclusion chromatography, following the method of Lee et al. [4]. Briefly, 95% cold ethanol (EtOH) was added to the KRG concentrate. The solution was treated with α-amylase and amyloglucosidase, and then the enzyme reaction was quenched before 95% EtOH was added to precipitate the polysaccharide fraction again. The lyophilized polysaccharide was treated with pectinesterase and then hydrolyzed with polygalacturonase. The lysates were eventually prepared as dried NFP by the method described after lyophilization. The NFP was analyzed to determine its contents of ginsenosides, arginine-fructose-glucose (AFG) and acidic polysaccharide (AP). In general, acidic polysaccharides consist of acidic sugars, such as galacturonic acid, glucuronic acid, and mannuronic acid, and are known to have greater activity than neutral polysaccharides [47]. Among the acidic polysaccharides of KRG reported so far, it is the active acidic polysaccharide that contains galacturonic acid in its constituent sugars. Thus, indirect quantification, using a colorimetric method and carbazole-sulfuric acid, is generally used for polysaccharide analysis [48,49,50,51,52]. The analysis of AFG, AP, and ginsenosides was performed as previously described [53]. No ginsenosides were detected in the NSF. The AFG and AP contents of the NFP were 11.63 and 438.08 mg/g, respectively. Comparing the previous AP results of KRG, the AP content was approximately fourfold higher than that of KRG [53].

### 4.3. Global Proteomic Profiling Analysis

#### 4.3.1. Animal Model

All animal experiments were conducted with the approval of the Institutional Animal Care and Use Committee of the Korean Ginseng Research Institute (Daejeon, Republic of Korea) following the Guide for the Care and Use of Laboratory Animals.

**Figure 6:**
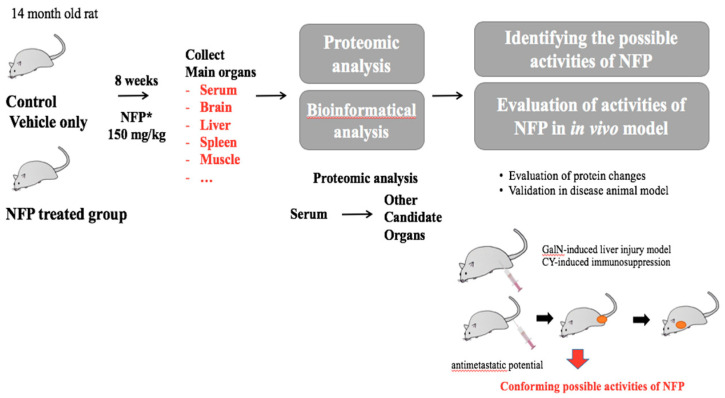
Schematic diagram of the experiments for the biological effects of NFP.

The male Sprague-Dawley rats (12 months old, Samtaco, Gyeonggi, Republic of Korea) were acclimatized at the Korean Ginseng Research Institute animal facility for 2 months before the experiment. They were housed at room temperature under humidity (36.2–56.3%) in a standard 12-h light/dark cycle. The 14-month-old male rats were randomly divided into 6 rats per group. The rats were administered 150 mg/kg NFP for 8 weeks. Only a vehicle was used for the negative control group (0 mg/kg) with a diet of animal chow and tap water. After dissection, the weights of the rat organs were measured, and the tissues were stored at −70 °C. (Figure 6).

#### 4.3.2. Serum Depletion

Serum samples were thawed on ice. The top three most abundant proteins were depleted using a MARS-MS3 (Agilent Technologies, Wilmington, DE) column. For this, the serum was filtered through 0.22-mm Spin-X filters and diluted (1:5) with a proprietary buffer A. The mixture was loaded onto the MARS-MS3 column on an Ultimate 3000 HPLC system (Dionex, Sunnyvale, CA, USA) with UV absorbance detector set at 214 nm. Unbound fractions containing the depleted serum were buffer-exchanged into 50 mM Tris-HCl (pH 8.0) and concentrated through ultrafiltration, using the Amicon Ultra-0.5 mL 3 kDa cutoff filter (Millipore, Darmstadt, Germany), to approximately 150 mL. Protein concentration was determined by Nanodrop (ThermoFisher Scientific, Bremen, Germany) measurement at A_280_nm.

#### 4.3.3. Protein Extraction

Rat spleen tissues were washed in 1X PBS and homogenized in a glass homogenizer with RIPA lysis buffer (Thermo Rockford, IL USA). The lysate was sonicated in ice for 2 min and centrifuged at 8000× *g* for 5 min at 4 °C twice. Rat liver and muscle tissues were homogenized using the CryoPrep™ system (Covaris, MA, USA). Approximately 1 mg of powdered, frozen tissue was transferred to 1.7-mL tubes and resuspended in radioimmunoprecipitation assay (RIPA) lysis buffer. After vortexing, each sample was sonicated in ice for 3 min and centrifuged at 3000× *g* for 5 min at 4 °C twice. The lysate was transferred to new 1.7-mL tubes and centrifuged at 10,000× *g* for 5 min. Bicinchoninic acid (BCA) quantification was performed with the Micro BCA Protein Assay Kit (Thermo Fisher Scientific).

#### 4.3.4. 1D SDS-PAGE Fractionation and In-gel Digestion

The 1D sodium dodecyl sulfate–polyacrylamide gel electrophoresis (SDS-PAGE) fractionation and in-gel tryptic digestion were conducted following the general protocol (Supplemental Method S1). Briefly, proteins were denatured and reduced in a lithium dodecyl sulfate (LDS) sample buffer with dithiothreitol (DTT), and then fractionated on a 4–12% gradient Bolt Bis-Tris gel (Invitrogen, MA, USA). The gel was stained with Instant Blue (Sigma-Aldrich, MO, USA). The gel lane was cut into 10 slices, then washed and destained. The proteins in the gels were reduced by DTT and alkylated with iodoacetamide (IAA). The gel particles were saturated with 12.5 ng/μL trypsin (Promega, Madison, WI, USA) for protein digestion. The digested peptides were extracted after incubation with 10% formic acid (FA). The extracted peptides were dried under a concentrator and stored at −20 °C until the liquid chromatography with tandem mass spectrometry (LC-MS/MS) analysis.

#### 4.3.5. Protein Identification Using LC-MS/MS Analysis

The dried peptide mixture was resuspended in solvent A (0.1% formic acid). The general instrumental analysis condition followed the method previously described [6]. The serum and spleen peptide samples were separated by online reversed-phase chromatography using a Thermo Scientific EASY-*n*LC 1200 ultra-high performance liquid chromatography (UHPLC) equipped with a reversed-phase peptide trap Acclaim PepMap^TM^ 100 autosampler (75 μm inner diameter, 2 cm length; Thermo Scientific) and a reversed-phase analytical column PepMap^TM^ RSLC C18 (75 μm inner diameter, 15 cm length, 3 μm particle size; Thermo Scientific). After separation, this was followed by electrospray ionization at a flow rate of 300 nL/min^−1^. The samples were eluted using a split gradient of 3–50% solution B (80% ACN with 0.1% FA) for 60 min, and 50–80% solution B for 10 min, followed by a column wash at 100% solution B for 10 min. The chromatography system was coupled with an Orbitrap Fusion mass spectrometer operated in a data-dependent mode, with a 120,000-resolution MS1 scan (375–1500 *m/z*), an Automated Gain Control (AGC) target of 5e^5^, and a max injection time of 50 ms. Peptides above the 5e^3^ threshold and charges 2–7 were selected for fragmentation with dynamic exclusion after a 15-second scan at 10 ppm tolerance.

The liver peptide samples were analyzed using a Q-Exactive mass spectrometer (Thermo Fisher Scientific, Bremen, Germany) equipped with a nano-UHPLC Dionex system (Thermo Fisher Scientific) and an Easy-Spray Source (Thermo Fisher Scientific). Every peptide sample was separated with a linear gradient from 2% to 35% or 40% Solvent B (0.1% FA in ACN), at a flow rate of 300 nL/min over 100 min. Precursor ions were acquired in the range of 350–1400 *m/z* under 70 k resolution (at 200 *m/z*), and the top 10 precursors were subjected to LC-MS/MS analysis at 2 Th (Thomson) of precursor isolation width. A higher-energy collisional dissociation with 27% collision energy, with a target value of 1E^6^ ions determined by automatic gain control, 60 ms maximum injection time and 17.5 k resolution at 200 *m/z*, was applied for LC-MS/MS analysis. 

#### 4.3.6. Data Search, Statistical Analysis, and Bioinformatic Analysis

All searches and subsequent bioinformatics analyses were carried out based on the method previously described [6]. Briefly, collected raw files were converted into mzXML files. The peptides were assigned by the SEQUEST algorithm (Thermo Fisher Scientific) against the decoy UniProt database (UniProt, http://www.uniprot.org/). The data set was entered into the R program (version 3.5.3) with a power law global error model (PLGEM, version 1.54.1) used to determine the signal-to-noise ratio (S/N) and *p*-value [54]. Based on PLGEM, the annotation of protein cellular localization and the evaluation of biological function were performed using the Ingenuity Pathway Analysis (IPA; Ingenuity Systems; Redwood City, CA, USA) database and the Database for Annotation, Visualization and Integrated Discovery (DAVID, https://david-d.ncifcrf.gov/).

### 4.4. IgM Antibody-Producing Cells in the Spleen 

Six-week-old male Bagg Albino (BALB/c) mice were purchased from DBL (Chungbuk, Republic of Korea). Animal experiments were conducted with the same housing conditions described above. The BALB/C mice were divided into 5 groups of 6 mice each to quantify the IgM-producing plasma cells. The blank group (normal) and the cyclophosphamide (CY)-treated group (CY control) were administered distilled water, while the NFP-treated groups were orally administered NFP at 75, 150, and 300 mg/kg for 10 consecutive days. Four days before the autopsy, immune reactions in mice were induced by injecting them with sheep red blood cells (SRBCs) intraperitoneally. The procedure for IgM antibody-producing cells follows the method of Hyun, et al. [55]. Briefly, the SRBCs had been refrigerated and used within 2 weeks of storage. After washing the SRBCs with Earle’s Balanced Salt Solution (EBSS) via centrifugation, their concentrations were adjusted using EBSS to 5 × 10^8^ cells/mL (5%). For the immune responses of the mice, the mice were intraperitoneally administered 50 mg/kg CY for immunosuppression, and then the mice were sacrificed to remove the spleen after three days. The spleen was placed in ice-cooled EBSS, and lightly ground using a sterile syringe, and then filtered through a mesh for subsequent centrifugation. After removing the supernatant, the procedure was repeated. The suspension was taken and mixed with EBSS to produce a five-fold diluted cell suspension. For the suspension of the SRBCs, the refrigerated cells were washed three times with EBSS by centrifugation (300× *g*, 10 min, 4 °C) immediately before use. A 0.5% agar solution was prepared by first dissolving the agar in EBSS containing 15 mM 4-(2-hydroxyethyl)piperazine-1-ethanesulfonic acid buffer solution. Once boiled, the solution was kept at 48 °C in a water bath, A 350 μL agar solution, 100 μL splenic cell solution, 25 μL SRBCs suspension, and 25 μL guinea pig complement were added and mixed thoroughly to a round-bottom tube (12 × 75 mm). Then, 200 μL of aliquot was transferred to a petri dish. After approximately 20 min, when the agar hardened, the cultivation was carried out in a 37 °C, 5% CO_2_ incubator for 4 h to induce plaque formation. The formed plaques were counted using an optical microscope (IX-81, Olympus, Tokyo, Japan) at a 10× magnification. The cell count in the splenic cell solution was then estimated, after which the result was converted to the number of plasma cells per 1 × 10^6^ spleen cells. 

### 4.5. Experimental Murine Lung Metastasis 

The experimental lung metastasis was performed according to Yun et al.’s method [56]. Six 6-week-old C57BL male mice (Koatech, Pyungtaek, Korea) were acclimated to laboratory conditions for 2 weeks. The mice were randomly assigned to 4 groups (*n* = 6/group), all of which were intravenously injected with B16F10 cells (ATCC) at a density of 2 × 10^6^ cells/mouse through the tail. The mice in the first group, as vehicle control, were treated with saline once per day by oral gavage. The other mice groups were orally administered with NFP at doses of 75, 150, and 300 mg/kg in saline once per day for 2 weeks. On the 14th day, mice were sacrificed by CO_2_ asphyxiation, and their lungs were dissected to count the number of metastatic colonies.

### 4.6. Galactosamine (GalN)-Induced Rat for Liver Injury Model

Clean male Sprague-Dawley rats were provided by Daehan Biolink Co. Ltd. (Eumsung, Korea) for the present study. The housing condition was the same as the housing condition described above. Rats were randomly assigned into six groups of equal size (6 rats/group) after a one-week adaptation period to environmental conditions. These six groups, the normal control group, the GalN group, the positive control group with milk thistle extract (100 mg/kg), and the three NFP groups, were orally gavaged with 25, 50, and 100 mg/kg once a day for 7 consecutive days. Then, GalN was injected 2 h after the final administration to induce liver injury and investigate the reduction of oxidative stress. The normal control and GalN groups received an equal amount of saline. At 24 h after the administration of the inducers and vehicles, rats were anesthetized, and blood and livers were collected. Serum samples were separated by centrifugation at 2000× *g* for 10 min. One part of the dissected liver was kept in 10% formalin for histopathological examination. At the same time, another part was immediately put in liquid nitrogen and kept at –90 °C to determine oxidative stress markers.

#### 4.6.1. Evaluation of Liver Function and Oxidative Stress Markers

Serum samples were tested for aspartate transaminase (AST) and alanine transaminase (ALT). Superoxide dismutase (SOD), glutathione peroxidase (GPx), and malondialdehyde (MDA), a lipid peroxidation product, were analyzed using a commercial diagnostic kit (Cayman Chemical, MI, USA) according to the manufacturer’s instruction. Liver enzymes, such as serum AST and ALT, levels were measured using a Hitachi Automatic Analyzer 7600 (Haiachi, Tokyo, Japan).

#### 4.6.2. Histologic Examination

The isolated liver tissues were fixed using 10% neutral formaldehyde. Specimens were left for an overnight wash, dehydrated in graded ethanol, cleared in xylene, and immersed in paraffin. Then, 5-micron pieces were sectioned using a rotary microtome, and slides were then dewaxed using xylene, hydrated with ethanol. The sections were stained with hematoxylin and eosin (H&E) for histopathological evaluation. The general microscopical structure of hepatic tissues was examined under a light microscope (Olympus, Shinjuku City, Japan).

## Figures and Tables

**Table 1:** Selected biosignature candidate proteins identified in the serum of the aged rat treated with non-saponin fractions with rich polysaccharide (NFP).

Up/Down	Identified Proteins	Gene	Accession Number	Functional Annotation Clustering	STN	*p*-Value
Upregulated Protein	Serine protease inhibitor A3N	Serpina3n	P09006	Secreted protein, cellular response to interleukin-6, phosphoprotein	8.58	0.00094
	Ac1873	Fga	Q7TQ70	Glycoprotein, cellular response to interleukin-6, acute-phase response, structural molecule activity	6.65	0.00206
	Alpha-2-macroglobulin	LOC100911545	M0R9G2		6.56	0.00211
	Fibrinogen beta chain	Fgb	P14480	Secreted protein, structural molecule activity	6.43	0.00251
	Retinol-binding protein 4	Rbp4	P04916	Disulfide bond	4.78	0.00439
	Aa1249	Crp	Q7TMA9	Cellular response to interleukin-6, acute-phase response, glycoprotein, secreted protein, disulfide bond	4.30	0.00533
	Murinoglobulin-1	Mug1	Q03626	Acute-phase response, glycoprotein, disulfide bond	4.24	0.00648
	Keratin, type I cytoskeletal 17	Krt17	Q6IFU8	Structural molecule activity, phosphoprotein	4.09	0.00711
	Isoform Gamma-A of Fibrinogen gamma chain	Fgg	P02680-2		3.68	0.00931
	Myoglobin	Mb	Q9QZ76	Phosphoprotein	3.44	0.01052
	Keratin, type II cytoskeletal 5	Krt5	A0A0G2K509	Structural molecule activity, phosphoprotein	3.21	0.01164
	Keratin, type II cytoskeletal 5	Krt5	Q6P6Q2	Structural molecule activity, phosphoprotein	3.14	0.01193
	Keratin, type I cytoskeletal 10	Krt10	Q6IFW6	Structural molecule activity, phosphoprotein	3.07	0.01207
	Glutathione S-transferase Mu 1	Gstm1	P04905	Glutathione metabolism, thioredoxin-like fold, metabolism of xenobiotics by cytochrome P450	2.67	0.01427
	Glutathione S-transferase alpha-3	Gsta3	P04904	Glutathione metabolism, thioredoxin-like fold, metabolism of xenobiotics by cytochrome P450	2.58	0.01500
	Carbonic anhydrase 3	Ca3	P14141	Phosphoprotein	2.55	0.01516
	Fatty acid-binding protein, adipocyte	Fabp4	P70623	Phosphoprotein	2.55	0.01516
	Ribonuclease UK114	Hrsp12	P52759	Phosphoprotein	2.39	0.01642
	Keratin, type I cytoskeletal 14	Krt14	Q6IFV1	Structural molecule activity, phosphoprotein	2.36	0.01667
	LOC367586 protein	LOC367586	Q5M7V3		2.33	0.01697
	Glutathione S-transferase Mu 2	Gstm2	P08010	Glutathione metabolism, thioredoxin-like fold, metabolism of xenobiotics by cytochrome P450	2.09	0.01979
	Fibrinogen-like protein 1	Fgl1	Q5M8C6	Secreted protein	1.85	0.02379
Downregulated Protein	Apolipoprotein A-IV	Apoa4	P02651	Secreted protein	−1.91	0.02326
	Hemopexin	Hpx	P20059	Secreted protein	−1.95	0.02255
	Coactosin-like protein	Cotl1	B0BNA5	Actin binding	−1.97	0.02220
	Transforming growth factor beta-1	Tgfb1	P17246	Hepatitis B, secreted protein	−1.98	0.02218
	14-3-3 protein theta	Ywhaq	P68255	Hepatitis B, viral carcinogenesis, actin binding	−2.09	0.02070
	14-3-3 protein eta	Ywhah	P68511	Hepatitis B, viral carcinogenesis, actin binding	−2.17	0.01986
	Myl6 protein	Myl6	B2GV99		−2.19	0.01966
	Vinculin	Vcl	P85972	Platelet aggregation, cell–cell adhesion, actin binding	−2.27	0.01831
	Hemoglobin subunit beta-1	Hbb	P02091	Platelet aggregation, secreted protein	−2.29	0.01812
	Tropomyosin alpha-4 chain	Tpm4	P09495	Actin binding	−2.36	0.01740
	Cofilin-1	Cfl1	P45592	Actin binding	−2.37	0.01739
	Afamin	Afm	G3V9R9	Actin binding, secreted protein	−2.46	0.01643
	Adenylyl cyclase-associated protein 1	Cap1	Q08163	Actin binding	−2.57	0.01562
	Profilin-1	Pfn1	P62963	cell-cell adhesion, actin binding	−2.69	0.01463
	14-3-3 protein zeta/delta	Ywhaz	P63102	Viral carcinogenesis, actin binding	−3.09	0.01227
	Transgelin-2	Tagln2	Q5XFX0	Cell–cell adhesion	−3.55	0.01006
	Filamin alpha	Flna	C0JPT7	Platelet aggregation	−4.18	0.00688
	Protein Tln1	Tln1	G3V852	Platelet aggregation	−4.98	0.00399
	Actin, cytoplasmic 1	Actb	P60711	Platelet aggregation, actin binding	−5.39	0.00301
	Apolipoprotein C-III	Apoc3	A0A0G2K8Q1	Secreted protein	−5.76	0.00292

Representatives of selected top upregulated or downregulated proteins from the serum of the NFP-treated aged rat model. The intensities of proteins were calculated using the power law global error model (PLGEM) within a triplicate analysis. STN: Signal to noise, *p*-value by PLGEM analysis.

**Table 2:** Identified possible functions of NFP in the serum of the rat treated with NFP by Ingenuity Pathway Analysis (IPA).

Up/Down	Total Function	Cancer-Specific	Except Cancer
**Upregulated**	**Quantity of cells**	**Concentration of lipid, fatty acid**	**Activation of phagocytes, macrophages**
	- myeloid cells	**Concentration of lipopolysaccharide**	**Quantity of cells**
	- blood cells	**Conjugation of glutathione**	- myeloid cells
	**Quantity of cells**	**Cellular infiltration by leukocytes**	- leukocytes
	**Activation of cells**		- blood cells
	- phagocytes		**Quantity of connective tissue**
	- macrophages		
	**Quantity of connective tissue**		
**Downregulated**	**Cancer**	**Cancer Type**	**Apoptosis, cell death**
	- Non-Hodgkin lymphoma	- Abdominal carcinoma, malignant solid tumor	- microvascular endothelial cells
	- Non-small cell lung carcinoma	- Nonhematologic malignant neoplasm, lung cancer	**Damage of epithelial, endothelial tissue**
	- Binding of tumor cell lines	- Liver cancer	**Apoptosis, cell death**
	- Lung carcinoma	- Non-Hodgkin lymphoma	- Endothelial cells
	**Brain**	- Non-melanoma solid tumor	**Damage of nervous system**
	- Tauopathy	- Extracranial solid tumor	**Production of reactive oxygen species**
	- Dementia	- Digestive system cancer	**Damage of endothelial cells**
	- Ischemia of brain	- Tumorigenesis of epithelial neoplasm	**Synthesis of reactive oxygen species**
	**Viral Infection**	**Anticancer function**	**Necrosis of epithelial tissue**
	**Chronic kidney disease**	- Metastasis of breast cancer cell lines	**Binding of neutrophils**
	**Neuromuscular disease**	- Cell spreading of tumor cell lines	**Organismal death**
	**Thrombosis**	- Development of malignant tumor	**Adhesion of myeloid cells**
	- Thrombosis of vein	- Incidence of tumor	
	- Coagulation of blood	- Angiogenesis	
	**Fibrogenesis**		
	**Synthesis of reactive oxygen species**		

**Table 3:** Selected biosignature candidate proteins identified in the spleen and liver of the aged rat treated with NFP.

Up/Down	Top 60 Spleen Proteins	Gene	Accession Number	Signal to Noise (STN)	*p*-Value	Top 60 Liver Proteins	Gene	Accession Number	STN	*p*-Value
Upregulated Protein	Spectrin, alpha, erythrocytic 1	Spta1	D4A678	32.1	0.00002	Hemoglobin subunit alpha 1/2	Hba1	P01946	16.9	0.00000
	Myosin 9	Myh9	Q62812	22.7	0.00005	Globin a4	Hbb	A0A0G2JSW3	15.7	0.00000
	Spectrin beta chain	Sptb	A0A140UHX6	18.3	0.00005	Alpha-2-macroglobulin	A2m	P06238	7.7	0.00000
	Spectrin beta chain	Sptbn1	G3V6S0	14.7	0.00009	Beta-glo	Hbb-b1	Q6PDU6	6.8	0.00000
	Sialic acid-binding Ig-like lectin 1	Siglec1	A0A0G2K320	11.8	0.00016	Spectrin beta chain	Sptb	A0A140UHX6	6.3	0.00000
	DnaJ heat shock protein family (Hsp40) member C13	Dnajc13	D3ZN27	9.8	0.00016	Fatty acid-binding protein, liver	Fabp1	P02692	6.0	0.00000
	Myosin, heavy polypeptide 9, non-muscle	Myh9	G3V6P7	9.7	0.00016	Elongation factor 1-alpha 1	Eef1a1	P62630	5.7	0.00000
	Pre-mRNA processing factor 8, isoform CRAa	Prpf8	G3V6H2	9.3	0.00017	Betaine--homocysteine S-methyltransferase 1	Bhmt	A0A0G2JSK9	5.5	0.00000
	Spectrin alpha chain, non-erythrocytic 1	Sptan1	Q6IRK8	9.0	0.00017	Cystathionase (Cystathionine gamma-lyase)	LOC103691744	Q9EQS4	4.9	0.00000
	Stabilin 2/HARE	Stab2	E0X583	8.9	0.00017	Brefeldin A inhibited guanine nucleotide-exchange protein 2	Arfgef2	Q7TSU1	4.6	0.00000
	Microtubule-actin crosslinking factor 1	Macf1	A0A0G2K9T4	8.8	0.00017	Leucyl-tRNA synthetase	Lars	Q5PPJ6	4.3	0.00000
	Insulin-like growth factor 2 receptor	Igf2r	G3V824	8.4	0.00017	Peroxiredoxin 1	Prdx1	Q63716	4.3	0.00001
	Profilin-1	Pfn1	P62963	8.2	0.00017	Aldehyde oxidase 1	Aox1	F1LRQ1	4.1	0.00001
	Serine/threonine/tyrosine kinase 1	Styk1	D3ZHY0	8.1	0.00018	S-adenosylmethionine synthase	Mat1a	F1LZ34	4.1	0.00001
	Fatty acid synthase	Fasn	P12785	8.1	0.00018	AHNAK nucleoprotein	Ahnak	A0A0G2JU96	4.0	0.00001
	Telomerase protein component 1	Tep1	O08653	7.7	0.00019	Microtubule-associated protein	Map4	A0A0G2JW88	3.9	0.00001
	Filamin A	Flna	C0JPT7	7.5	0.00019	Sperm-associated antigen 9	Spag9	E9PSJ4	3.9	0.00001
	von Willebrand factor	Vwf	F1M957	7.3	0.00019	RAN-binding protein 2	Ranbp2	M0R3M4	3.7	0.00002
	Plectin	Plec	Q6S399	6.9	0.00019	Spectrin, alpha, erythrocytic 1	Spta1	D4A678	3.6	0.00002
	Nuclear pore membrane glycoprotein 210	Nup210	P11654	6.6	0.00020	Glutathione S-transferase alpha 3	Gsta3	P04904	3.6	0.00003
	Filamin B	Flnb	A0A0G2JXT8	6.4	0.00020	Isoform 2 of E3 ubiquitin-protein ligase TRIP 12	Trip12	F1LP64-2	3.5	0.00003
	Isoform 3 of electrogenic sodium bicarbonate cotransporter 1	Slc4a4	Q9JI66-3	6.4	0.00020	Tyrosine-protein phosphatase non-receptor type 23	Ptpn23	F1M951	3.5	0.00003
	Vacuolar protein sorting 13 homolog C	Vps13c	D4A4K4	6.2	0.00021	Ubiquitin-specific peptidase 24	Usp24	F1LSM0	3.4	0.00004
	ATP-binding cassette subfamily A member 1	Abca1	F1LNL3	5.8	0.00025	Alpha-1-inhibitor 3	A1i3	P14046	3.4	0.00004
	MHC class I alpha chain (Fragment)		O02953	5.7	0.00025	Phosphoserine aminotransferase	Psat1	Q68FU2	3.4	0.00004
	Hemoglobin subunit alpha 1/2	Hba1	P01946	5.5	0.00029	Activating signal cointegrator 1 complex subunit 3	Ascc3	A0A0A0MY43	3.4	0.00004
	Alpha-2-macroglobulin	A2m	P06238	5.2	0.00030	Myosin 11	Myh11	E9PTU4	3.4	0.00004
	Mx2		J7JVB9	5.1	0.00030	Similar to KIAA0368	Ecpas	F1M446	3.3	0.00004
	3-ketoacyl-CoA thiolase, mitochondrial	Acaa2	A0A0G2K642	4.7	0.00037	Glutamyl-prolyl-tRNA synthetase	Eprs	A0A0G2JZI2	3.3	0.00004
	Structural maintenance of chromosomes protein	Smc4	F1MAD9	4.7	0.00037	Eukaryotic translation initiation factor 4 gamma, 1	Eif4g1	D3ZU13	3.3	0.00004
Downregulated Protein	Fibrillin 1	Fbn1	G3V9M6	−24.1	0.00005	Plectin	Plec	Q6S395	−10.2	0.00000
	Collagen type IV alpha 1 chain	Col4a1	F1MA59	−18.8	0.00005	Carbamoyl-phosphate synthase [ammonia], mitochondrial	Cps1	P07756	−10.2	0.00000
	Collagen type IV alpha 2 chain	Col4a2	F1M6Q3	−18.8	0.00005	Fatty acid synthase	Fasn	P12785	−8.6	0.00000
	Laminin subunit alpha 5	Lama5	F1MAN8	−13.3	0.00016	Desmoplakin	Dsp	F1LMV6	−6.4	0.00000
	Laminin subunit gamma 1	Lamc1	F1MAA7	−13.3	0.00016	RCG34348, isoform CRAa	Krt33a	Q6IFW1	−6.2	0.00000
	Uncharacterized protein		F1LTJ5	−13.2	0.00016	Spectrin alpha chain, non-erythrocytic 1	Sptan1	A0A0G2JZ69	−5.9	0.00000
	Collagen type VII alpha 1 chain	Col7a1	D3ZE04	−13.1	0.00016	Amylo-1, 6-glucosidase, 4-alpha-glucanotransferase (glycogen debranching enzyme, glycogen storage disease type III) (Predicted), isoform CRAa	Agl	D4AEH9	−5.7	0.00000
	Fibronectin	Fn1	A0A096P6L8	−12.7	0.00016	Filamin B	Flnb	A0A0G2JXT8	−5.2	0.00000
	Laminin subunit beta 2	Lamb2	M0R6K0	-12.0	0.00016	Lysophospholipase-like 1	Lyplal1	D3ZFS7	-4.9	0.00000
	RCG34610, isoform CRAc	Srsf1	D4A9L2	−10.8	0.00016	Peroxisomal bifunctional enzyme	Ehhadh	P07896	−4.3	0.00000
	Collagen type VI alpha 2 chain	Col6a2	F1LNH3	−10.8	0.00016	Glycogen phosphorylase, liver form	Pygl	P09811	−4.2	0.00000
	Collagen alpha-1(I) chain	Col1a1	P02454	−8.8	0.00017	Ornithine carbamoyltransferase, mitochondrial	Otc	P00481	−4.2	0.00000
	Ferritin light chain 1	Ftl1	P02793	−8.6	0.00017	Endoplasmin	Hsp90b1	A0A0A0MY09	−4.2	0.00000
	Transglutaminase 2, C polypeptide	Tgm2	Q6P6R6	−7.8	0.00019	Mitochondrial pyruvate carrier 1	Mpc1	P63031	−4.1	0.00000
	Junction plakoglobin	Jup	Q6P0K8	−7.5	0.00019	Aldehyde dehydrogenase, mitochondrial	Aldh2	F1LN88	−4.1	0.00001
	Collagen, type I, alpha 2	NEWGENE_621351	A0A0G2K5E8	−7.3	0.00019	Spectrin beta chain	Sptbn2	F1MA36	−4.0	0.00001
	Nephronectin	Npnt	A0A0G2JW46	−7.2	0.00019	D-beta-hydroxybutyrate dehydrogenase, mitochondrial	Bdh1	P29147	−3.9	0.00001
	Transformer-2 protein homolog beta	Tra2b	P62997	−7.1	0.00019	Syntaxin 3	Stx3	Q08849	−3.7	0.00002
	Collagen type VI alpha 1 chain	Col6a1	D3ZUL3	−7.0	0.00019	Maleylacetoacetate isomerase	Gstz1	P57113	−3.7	0.00003
	LDL receptor-related protein 1	Lrp1	G3V928	−6.5	0.00020	Dimethylaniline monooxygenase [N-oxide-forming]	Fmo5	A0A0G2JSQ2	−3.6	0.00003
	Latent-transforming growth factor beta-binding protein 1	Ltbp1	D3ZAA3	−6.4	0.00020	Membrane-associated progesterone receptor component 1	Pgrmc1	P70580	−3.6	0.00003
	Ferritin		M0R5T8	−6.4	0.00020	Inositol 1,4,5-trisphosphate receptor type 2	Itpr2	P29995	−3.6	0.00003
	Serine/arginine-rich splicing factor 2	Srsf2	Q6PDU1	−6.2	0.00021	Cytochrome P450, family 2, subfamily c, polypeptide 7	Cyp2c7	Q4QQW7	−3.4	0.00004
	RCG61762, isoform CRA_d	Srsf7	D4A720	−5.7	0.00025	Paternally-expressed 3	Peg3	D4AB33	−3.4	0.00004
	Desmoplakin	Dsp	F1LMV6	−5.7	0.00025	Elongation factor 2	Eef2	P05197	−3.3	0.00005
	Nidogen 1	Nid1	F1LM84	−5.6	0.00026	3-Ketoacyl-CoA thiolase, peroxisomal	Acaa1a	P21775	−3.3	0.00005
	Eosinophilroxidase	Epx	D3ZSY4	−5.2	0.00030	Vacuolar protein sorting 13 homolog A	Vps13a	D4A899	−3.2	0.00006
	Histone H2A type 2A	Hist2h2aa3	P0CC09	−5.2	0.00030	Pyruvate carboxylase, mitochondrial	Pc	P52873	−3.2	0.00007
	Histone H4	Hist1h4b	P62804	−5.1	0.00031	Spectrin beta chain	Sptbn1	G3V6S0	−3.1	0.00007
	Pre-mRNA processing factor 40 homolog A (Yeast) (Predicted)	Prpf40a	D3ZJ92	−4.9	0.00037	ATP-binding cassette, subfamily A (ABC1), member 8a	Abca8a	D3ZCF8	−3.1	0.00007

Representatives of selected upregulated or downregulated proteins from the serum of the NFP-treated aged rat model. The intensities of proteins were calculated by PLGEM within a triplicate analysis.

**Table 4:** Selected Database for Annotation, Visualization, and Integrated Discovery (DAVID) and IPA functional analysis of the identified proteins in the spleen and liver of the aged rat treated with NFP.

**A. Spleen**		
**Selected DAVID Analysis for Functional Annotation**	***p*-Value**	**Proteins**
Innate immune response	3.00E-01	C4bpa, Fga, Mx1, Styk1
Biosynthesis of antibiotics	4.00E-02	Acaa2, Adsl, Aldoc, Eno1, Hadhb, Pgd, Prps2
ATP-binding	7.20E-02	Abca1, Ddx5, Rock2, Ilk, Mthfd1, Myh9, Myh9l1, Prps2, Prkacb, Smc1a, Tep1, Vcp
Amoebiasis	4.80E-06	Col1a1, Col4a1, Col4a2, Col4a5, NEWGENE_621351, Fn1, Lama5, Lamb2, Lamc1, Prkacb
**Main Disease and Biofunction**	**Activation z-Score**	**Related Proteins**
Cell movement of macrophages	1.951	Calr, Srsf1, Srsf3, Tf, Srsf2, Vcp
Cell movement of phagocytes	1.488	Plec, Col4a1, Fn1, Pfn1, Flna, Abca1, Lrp1, Stab1, Vwf, Col1a1, Ptprj, Alb
Cell proliferation of lymphoma cell lines	−2.4	Plec, Fn1, Pfn1, Flna, Stab1, Lrp1, Ptprj
Inflammation of body cavity	−1.455	Tf, Srsf2, Abca1, Fasn, Fga, Stab1, Col1a1, Ptprj, Myh9, Calr, Fn1, Epx, Alb
**B. Liver**		
**Selected DAVID Analysis for Functional Annotation**	***p*-Value**	**Genes**
Mitochondrion	3.10E-08	Decr1, Bdh1, Hibadh, Atp5a1, Atp5b, Cox2, Acsf2, Acsl1, Agxt2, Aldh2, Cps1, Dmgdh, Gstp1, Hspe1, Hadh, Mtor, Mpc1, Otc, Phb, Pc, Rmdn3, Sardh, Slc25a20
Glutathione metabolic process	1.20E-04	Gsta1, Gstm2, Gstp1, Gstz1, Gpx1, Hbb
Wound healing	6.30E-03	Aqp1, Dsp, Fn1, Gsn, Mtor, Tnc
**Main Disease and Bio Function**	**Activation z-Score**	**Related Proteins**
Liver lesion	−2.205	Itpr2, Gsta5, Atp1a1, Ass1, Cyp2e1, Tln2, Eef2, Aldob, Actb, Aox1, Lrp1, Eef1a1, Ugt2b7, Spag9, Cltc, Bdh1, Fabp7, Sptbn2, Fabp1, Pdia4, Apob, Gsn, Birc6, Lars, Decr1, Mat1a, Usp47, Flnb, Cps1, Comt, Hectd1, Fasn, Pc, Aqp1, Polr2b, Fah, Sptbn1, Eprs, Acsl1, Fn1, Aldh2, Arfgef2, Usp24, Gstp1, Trip11, Pzp, Cobll1, Arfgef1, Mtor, Pygl, Pklr, Ppia, Acaa1, Bhmt, Plec, Gstz1, Parp1, Copa, Cyp2c8, Cast, Araf, Mttp
Cell death of hepatocytes	−1.954	Sptbn1, Fah, Mtor, Gsn, Aldh2, Lrp1, Cyp2e1
Necrosis of liver	1.584	Sptbn1, Fah, Gstz1, Mtor, Gsn, Aldh2, Lrp1, Cyp2e1, Pzp
Inflammation of liver	−1.432	Fah, GSstz1, Apob, Ppia, Mat1a, Cyp2e1, Pzp, Mttp
Production of reactive oxygen species	−1.114	Mtor, Cyp2c8, Lrp1, Cyp2e1

Functional annotations of proteins in Table 4 were obtained from DAVID, and further detailed analysis was performed with IPA.

## Supplementary Materials

The following are available online. Method S1: 1D SDS-PAGE Fractionation and In-gel Digestion, Table S1: Serum protein list, Table S2: Serum protein list 99.9_95_min2, Table 3: Spleen protein list, Table S4. Liver protein list.
