# Supplementary material for: Biological Effects of Korean Red Ginseng Polysaccharides in Aged Rat Using Global Proteomic Approach
Source: Molecules. 2020 Jul 1;25(13):3019. doi: 10.3390/molecules25133019 (PMC7412055; doi:10.3390/molecules25133019)
Supplement: Supplementary file 1 [file molecules-25-03019-s001.zip › Supplementary Files/Figures/Fig. 2_revised.pptx]

## Slide 1
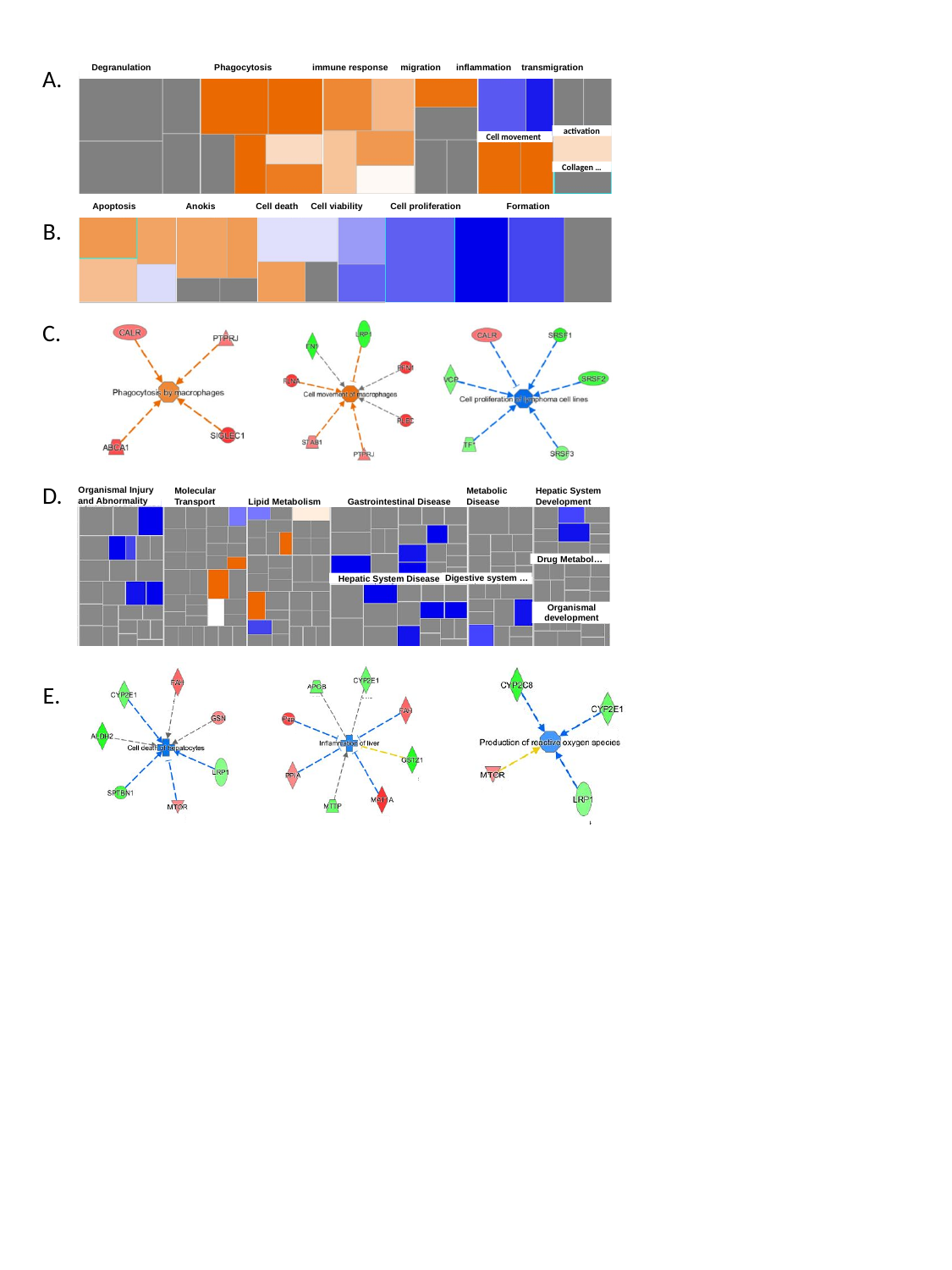

Degranulation Phagocytosis immune response migration inflammation transmigration
A.
activation
Cell movement
Collagen …
 Apoptosis Anokis Cell death Cell viability Cell proliferation Formation
B.
C.
D.
Organismal Injury and Abnormality
Metabolic Disease
 Molecular
 Transport
Hepatic System Development
Lipid Metabolism
Gastrointestinal Disease
Drug Metabol…
Digestive system …
Hepatic System Disease
Organismal
development
E.
