# Supplementary material for: Biological Effects of Korean Red Ginseng Polysaccharides in Aged Rat Using Global Proteomic Approach
Source: Molecules. 2020 Jul 1;25(13):3019. doi: 10.3390/molecules25133019 (PMC7412055; doi:10.3390/molecules25133019)

## Slide 1
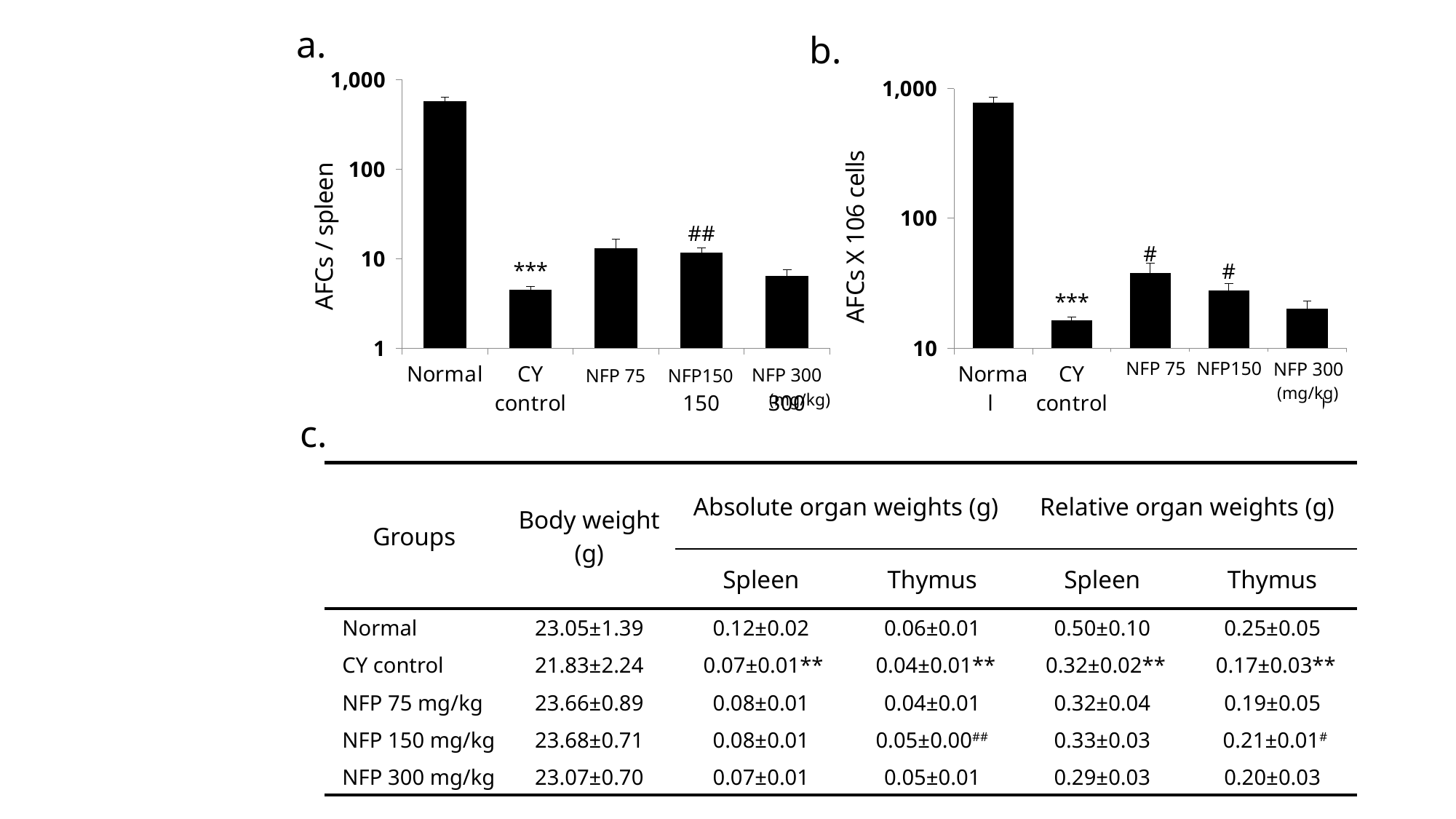

a.
b.
### Chart
| Category | |
|---|---|
| Normal | 772.7792429693839 |
| CY control | 16.270931464107168 |
| NSF 75 | 37.95306585540064 |
| NSF 150 | 27.910987079305407 |
| NSF 300 | 20.12487563533714 |
### Chart
| Category | |
|---|---|
| Normal | 576.0 |
| CY control | 4.5 |
| NSF 75 | 13.125 |
| NSF 150 | 11.625 |
| NSF 300 | 6.375 |NFP150
NFP 75
NFP 300
NFP 300
NFP 75
NFP150
(mg/kg)
(mg/kg)
c.
| Groups | Body weight (g) | Absolute organ weights (g) | | Relative organ weights (g) | |
| --- | --- | --- | --- | --- | --- |
| | | Spleen | Thymus | Spleen | Thymus |
| Normal | 23.05±1.39 | 0.12±0.02 | 0.06±0.01 | 0.50±0.10 | 0.25±0.05 |
| CY control | 21.83±2.24 | 0.07±0.01\*\* | 0.04±0.01\*\* | 0.32±0.02\*\* | 0.17±0.03\*\* |
| NFP 75 mg/kg | 23.66±0.89 | 0.08±0.01 | 0.04±0.01 | 0.32±0.04 | 0.19±0.05 |
| NFP 150 mg/kg | 23.68±0.71 | 0.08±0.01 | 0.05±0.00## | 0.33±0.03 | 0.21±0.01# |
| NFP 300 mg/kg | 23.07±0.70 | 0.07±0.01 | 0.05±0.01 | 0.29±0.03 | 0.20±0.03 |

## Slide 2
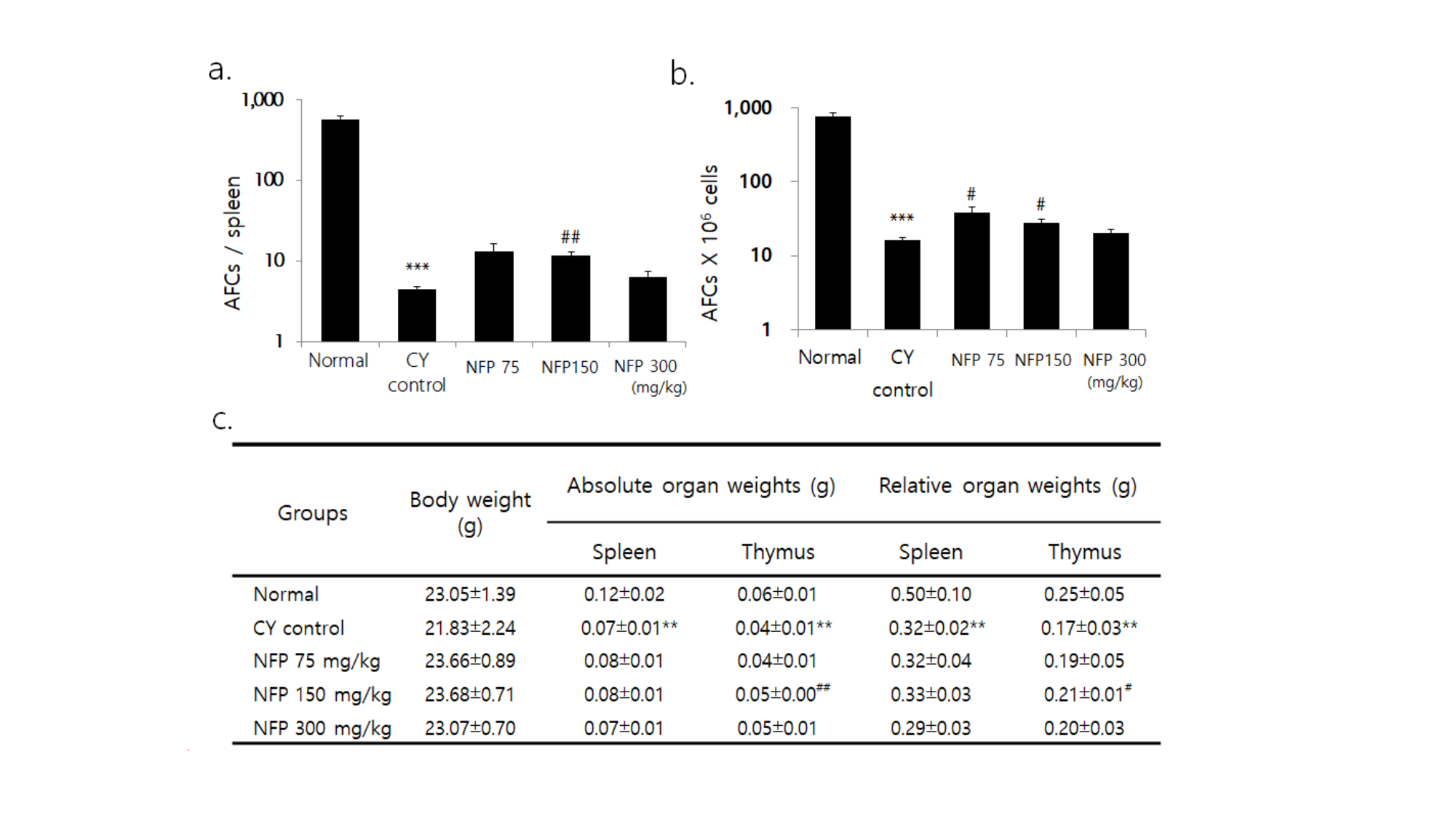

Supplement: Supplementary file 1 [file molecules-25-03019-s001.zip › Supplementary Files/Figures/Fig. 3_revised.pptx]
