# Supplementary material for: Biological Effects of Korean Red Ginseng Polysaccharides in Aged Rat Using Global Proteomic Approach
Source: Molecules. 2020 Jul 1;25(13):3019. doi: 10.3390/molecules25133019 (PMC7412055; doi:10.3390/molecules25133019)
Supplement: Supplementary file 1 [file molecules-25-03019-s001.zip › Supplementary Files/Figures/Fig. 4_revised.pptx]

## Slide 1
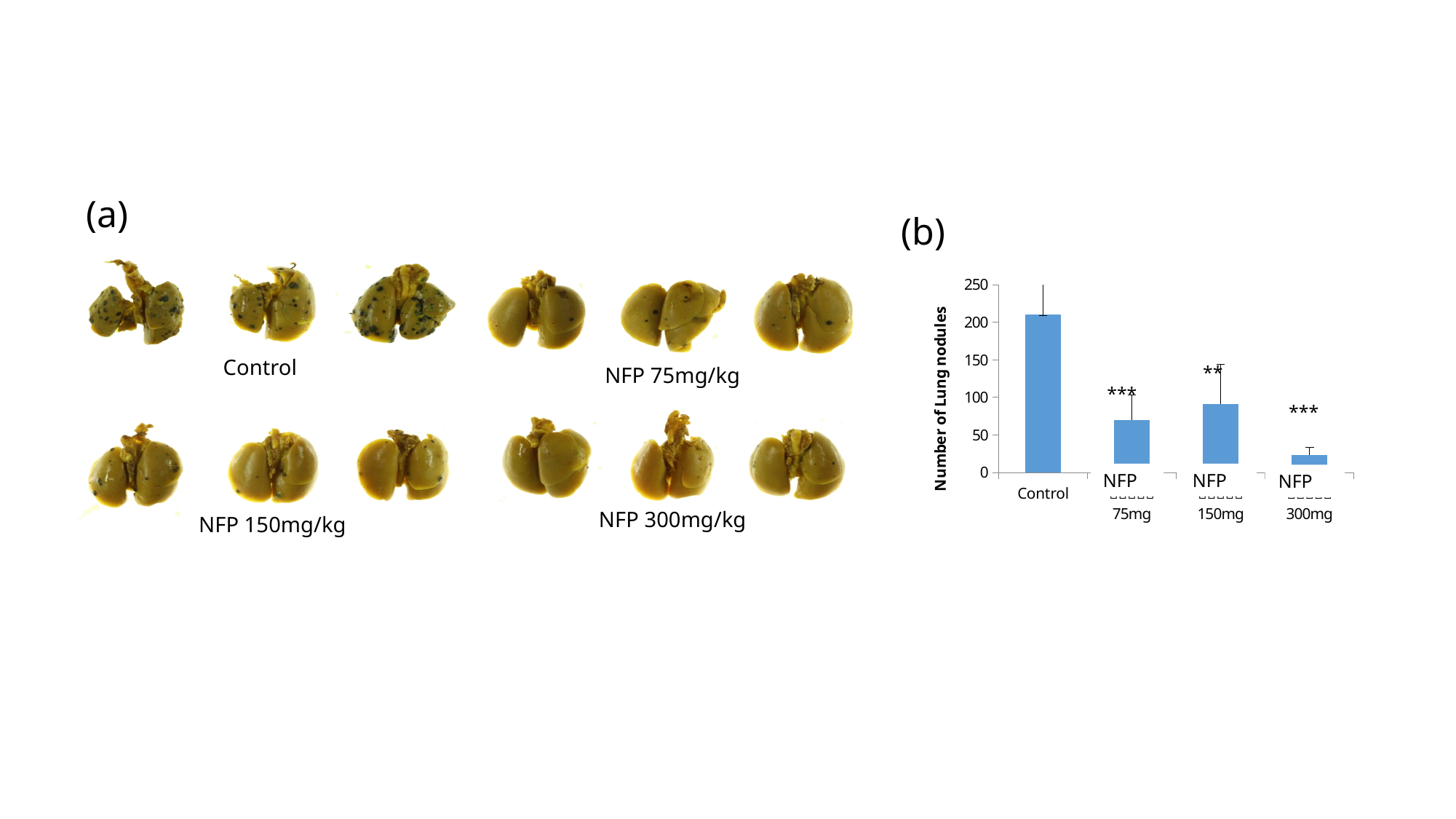

(a)
(b)
Control
NFP 75mg/kg
### Chart
| Category | |
|---|---|
| Control | 209.875 |
| 홍삼다당체
75mg | 69.77777777777774 |
| 홍삼다당체
150mg | 91.0 |
| 홍삼다당체
300mg | 23.28571428571427 |**
***
***
NFP 300mg/kg
NFP 150mg/kg
NFP
NFP
NFP
