# Supplementary material for: Biological Effects of Korean Red Ginseng Polysaccharides in Aged Rat Using Global Proteomic Approach
Source: Molecules. 2020 Jul 1;25(13):3019. doi: 10.3390/molecules25133019 (PMC7412055; doi:10.3390/molecules25133019)

## Slide 1
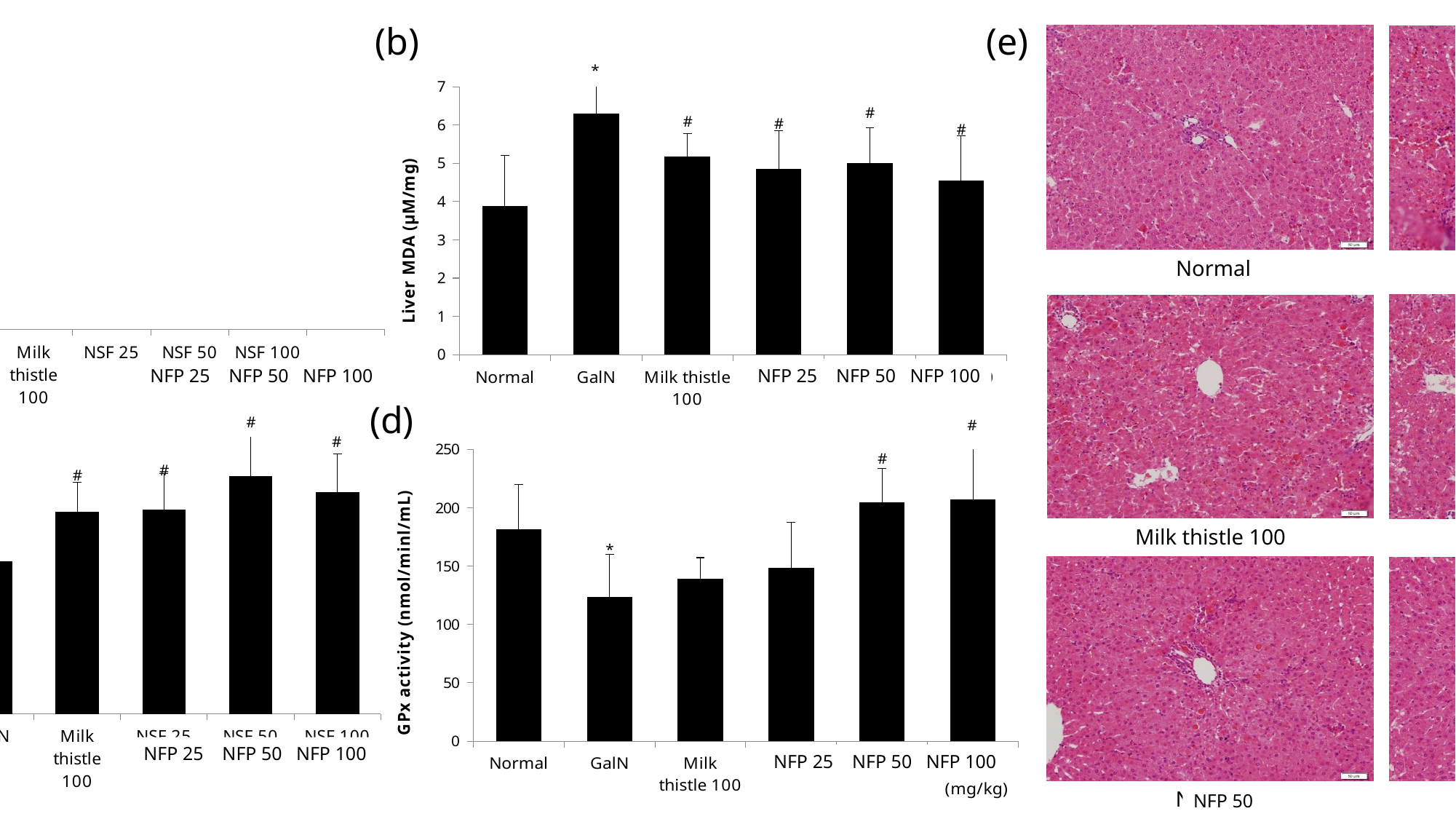

(a)
(b)
(e)
Normal
GalN
NSF 25
Milk thistle 100
NSF 50
NSF 100
### Chart
| Category | |
|---|---|
| Normal | 3.8824319672131153 |
| GalN | 6.288862295081969 |
| Milk thistle 100 | 5.170722950819673 |
| NSF 25 | 4.849865573770493 |
| NSF 50 | 5.010294262295083 |
| NSF 100 | 4.543592622950819 |
### Chart
| Category | |
|---|---|
| Normal | 151.83333333333334 |
| GalN | 10131.666666666666 |
| Milk thistle 100 | 7021.666666666667 |
| NSF 25 | 7043.333333333333 |
| NSF 50 | 9595.0 |
| NSF 100 | 7798.333333333333 |NFP 25 NFP 50 NFP 100
NFP 25 NFP 50 NFP 100
(c)
(d)
### Chart
| Category | |
|---|---|
| Normal | 84.63881196528894 |
| GalN | 49.59565250659967 |
| Milk thistle 100 | 65.61656406340123 |
| NSF 25 | 66.29863655243366 |
| NSF 50 | 77.37556857061386 |
| NSF 100 | 71.96690304181526 |
### Chart
| Category | |
|---|---|
| Normal | 181.63336351180342 |
| GalN | 123.4020850976418 |
| Milk thistle 100 | 138.9835482386586 |
| NSF 25 | 148.4711873136796 |
| NSF 50 | 204.8783926032116 |
| NSF 100 | 207.10953413829895 |NFP 25
NFP 25 NFP 50 NFP 100
NFP 25 NFP 50 NFP 100
NFP 50
NFP 100

## Slide 2
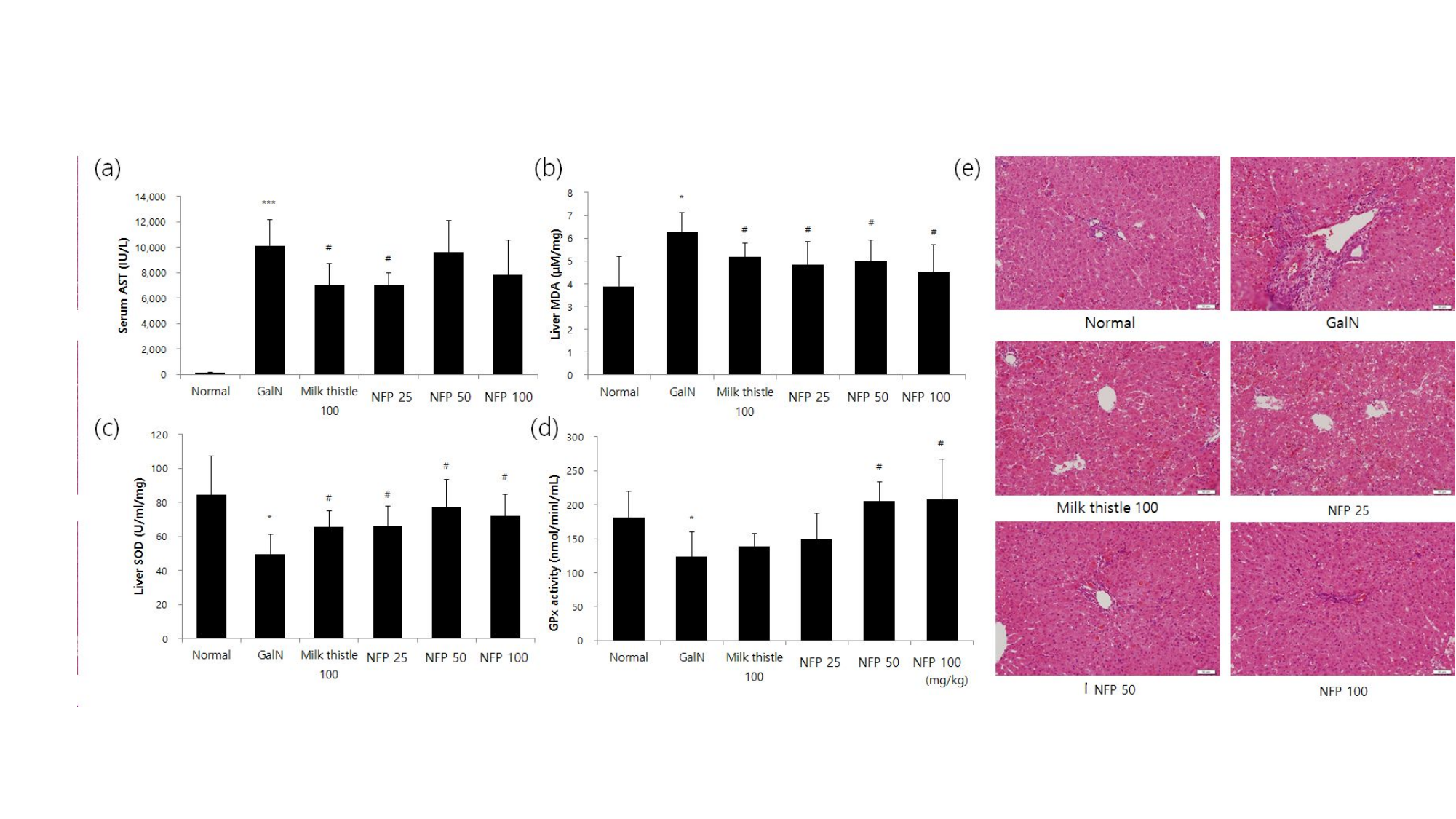

Supplement: Supplementary file 1 [file molecules-25-03019-s001.zip › Supplementary Files/Figures/Fig. 5_revised.pptx]
