# Supplementary material for: Biological Effects of Korean Red Ginseng Polysaccharides in Aged Rat Using Global Proteomic Approach
Source: Molecules. 2020 Jul 1;25(13):3019. doi: 10.3390/molecules25133019 (PMC7412055; doi:10.3390/molecules25133019)
Supplement: Supplementary file 1 [file molecules-25-03019-s001.zip › Supplementary Files/Supplemental-Method_1-and-2.docx]

[Supplementary Method 1]

Each gel lane was briefly cut into 10 slices and diced into 1 mm^3^ pieces on a glass plate using a clean scalpel. The excised gel pieces were then washed and destained three times for 20 min with a sufficient amount of 50% (v/v) ACN in 25 mM ammonium bicarbonate (NH_4_HCO_3_) (Sigma-Aldrich), and the supernatant was discarded in each step of the procedure. The gels were shrunk by 100% ACN and dried under a speed-vac concentrator, and the proteins in the gels were reduced by 25 mM DTT in 100 mM NH_4_HCO_3_ at 60℃ for 1 hr and alkylated with freshly prepared 55 mM iodoacetamide (IAA) (Sigma-Aldrich) in NH_4_HCO_3_ in the dark for 45 min. The gels were thoroughly washed twice, alternating between 50% ACN in 25 mM NH_4_HCO_3_ and 100 mM NH_4_HCO_3_ to eliminate the remaining trypsin interfering reagents, such as DTT and IAA. The gel particles were shrunk by 100% ACN and completely dried using the speed-vac concentrator. The shrunken pieces were saturated with 12.5 ng/μL trypsin (Promega, Madison, WI, USA) in 50 mM of NH_4_HCO_3_ on ice for 45 min before the excess trypsin solutions were removed, and 50 mM NH_4_HCO_3_ was added to cover the gel pieces. The proteins in the gel pieces were digested at 37℃ overnight, and the digested peptide solutions were collected into a clean tube. Afterward, 10% formic acid (FA) digested the peptides extracted after incubation for 15 min on ice, and another extraction step was performed after incubation with 50% (v/v) ACN / 0.1% (v/v) FA and 80% (v/v) ACN / 0.1% (v/v) FA serially. The extracted peptides were dried under a concentrator and stored at -20℃ before the LC-MS/MS analysis was performed.

[Supplementary Method 2]

Collected raw files were converted into the mzXML format through the Trans-Proteomic Pipeline (Seattle Proteomic Center, Seattle, WA, USA). The SEQUEST algorithm (Thermo Fisher Scientific) assigned each peptide against the decoy UniProt database (UniProt, http://www.uniprot.org/) under Sorcerer (Sage-N Research, Milpitas, CA, USA). All searches were carried out based on trypsin specificity, which allows two missed cleavages. The carbamidomethylation of cysteine and oxidation of methionine were fixed. Meanwhile, the search considered a precursor ion mass tolerance of 10 ppm, and the fragment ion mass tolerance was set at 1.0 Da. Peptide identifications were accepted if the PeptideProphet algorithm with Scaffold Q+ (Proteome Software, Portland, OR, USA) delta-mass correction (probability: protein ≧ 95%–99.9%, peptide ≧ 95%, 2 minimum peptides) could establish a greater than 95.0% probability. The data set was entered into the R program (version 3.5.3) with a power law global error model (PLGEM, version 1.54.1) and a statistical analysis software package (www.bioconductor.org) used to determine the signal-to-noise ratio (S/N) and P-value. Based on PLGEM, the protein cellular localization annotation and biological function evaluation were performed after uploading the S/N and P-value to the Ingenuity Pathway Analysis (IPA; Ingenuity Systems; Redwood City, CA, USA) database. A functional annotation analysis was performed using the Database for Annotation, Visualization, and Integrated Discovery (DAVID, https://david-d.ncifcrf.gov/) to identify the functional annotations represented by the differentially expressed proteins (DEPs).
