# Supplementary figures and images for: Biological Effects of Korean Red Ginseng Polysaccharides in Aged Rat Using Global Proteomic Approach
Source: Molecules. 2020 Jul 1;25(13):3019. doi: 10.3390/molecules25133019 (PMC7412055; doi:10.3390/molecules25133019)

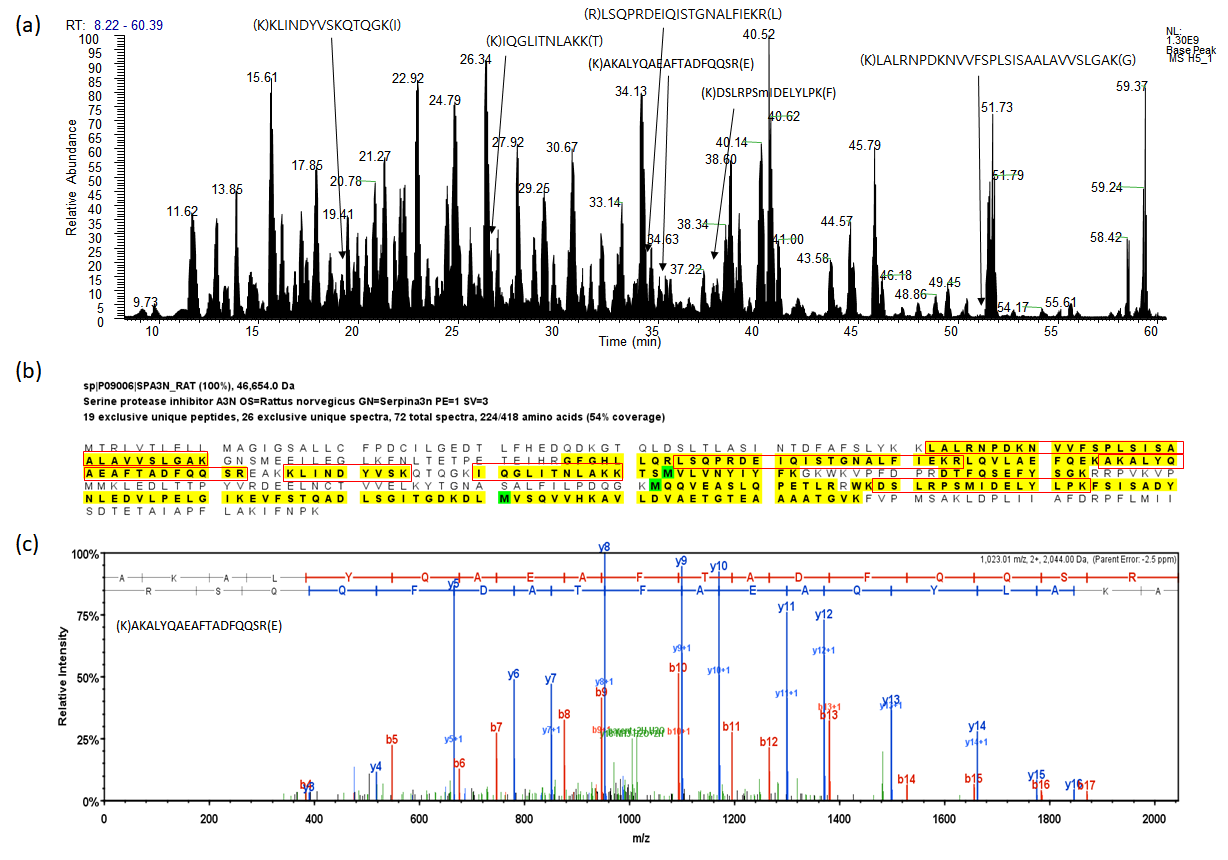

Supplement: Supplementary file 1 [file molecules-25-03019-s001.zip › Supplementary Files/Figures/Figure 1.tif]

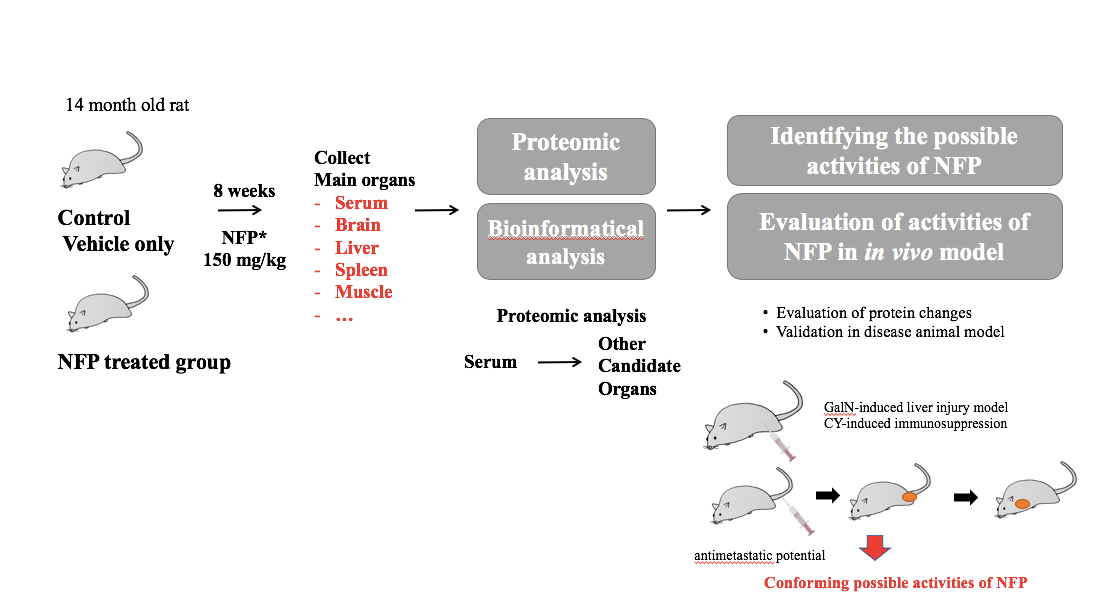

Supplement: Supplementary file 1 [file molecules-25-03019-s001.zip › Supplementary Files/Figures/Figure 6.tif]
